# The *Dkk3* gene encodes a vital intracellular regulator of cell proliferation

**DOI:** 10.1371/journal.pone.0181724

**Published:** 2017-07-24

**Authors:** Jack L. Leonard, Deborah M. Leonard, Scot A. Wolfe, Jilin Liu, Jaime Rivera, Michelle Yang, Ryan T. Leonard, Jacob P. S. Johnson, Prashant Kumar, Kate L. Liebmann, Amanda A. Tutto, Zhongming Mou, Karl J. Simin

**Affiliations:** 1 Department of Microbiology and Physiological Systems, University of Massachusetts Medical School, Worcester, Massachusetts, United States of America; 2 Program in Molecular Medicine, University of Massachusetts Medical School, Worcester, Massachusetts, United States of America; 3 Department of Biochemistry and Molecular Pharmacology, University of Massachusetts Medical School, Worcester, Massachusetts, United States of America; 4 Department of Molecular, Cell and Cancer Biology, University of Massachusetts Medical School, Worcester, Massachusetts, United States of America; 5 Department of Cell and Molecular Physiology, University of Massachusetts Medical School, Worcester, Massachusetts, United States of America; 6 Department of Cell and Developmental Biology, University of Massachusetts Medical School, Worcester, Massachusetts, United States of America; Florida International University, UNITED STATES

## Abstract

Members of the Dickkopf (*Dkk*) family of Wnt antagonists interrupt Wnt-induced receptor assembly and participate in axial patterning and cell fate determination. One family member, DKK3, does not block Wnt receptor activation. Loss of *Dkk3* expression in cancer is associated with hyperproliferation and dysregulated ß-catenin signaling, and ectopic expression of *Dkk3* halts cancer growth. The molecular events mediating the DKK3-dependent arrest of ß-catenin-driven cell proliferation in cancer cells are unknown. Here we report the identification of a new intracellular gene product originating from the *Dkk3* locus. This Dkk3b transcript originates from a second transcriptional start site located in intron 2 of the *Dkk3* gene. It is essential for early mouse development and is a newly recognized regulator of ß-catenin signaling and cell proliferation. Dkk3b interrupts nuclear translocation ß-catenin by capturing cytoplasmic, unphosphorylated ß-catenin in an extra-nuclear complex with ß-TrCP. These data reveal a new regulator of one of the most studied signal transduction pathways in metazoans and provides a novel, completely untapped therapeutic target for silencing the aberrant ß-catenin signaling that drives hyperproliferation in many cancers.

## Introduction

The Dickkopf family of secreted glycoproteins is composed of four members that first appeared in early metazoans as key regulators of the Wnt/ß-catenin signaling pathway [1–4]. Three family members DKK1, DKK2 and DKK4 bind to the LRP5/6 and Kremen subunits of the receptor [5] and prevent assembly of a functional Wnt receptor complex [6–8]. The remaining family member, DKK3, apparently evolved divergently [2, 9] and does not bind to LRP5/6 or modulate Wnt receptor assembly/signaling [10–13], even though it retains the two cysteine rich domains common to all family members [10]. Despite its inability to disrupt Wnt receptor binding, DKK3 is the best-known tumor suppressor in the family [11, 12]. DKK3 expression is frequently silenced in cancer, often by the hyper-methylation of CpG islands located in the *Dkk3* locus [13–15] and ectopic over-expression of DKK3 slows ß-catenin driven cancer cell proliferation *in vitro* [16–19]. To date, the molecular details of the mechanism DKK3 action remain elusive. Despite its presumed role in regulating ß-catenin driven cancer cell proliferation, targeted inactivation of the mouse *Dkk3* gene failed to provide a direct link between DKK3, the Wnt/ß-catenin signaling, and control of cell proliferation. The *Dkk3*^*tm1Cni*^ mutant mouse is viable, fertile, shows no ß-catenin signaling defects or any increase in cancer susceptibility [20] and failed to phenocopy other Dickkopf deletion mutants [21–25] or deletion mutants of individual components the Wnt/ß-catenin pathway [26–32].

In this study, we show that the *Dkk3* gene encodes a second vital intracellular isoform, DKK3b, that inhibits hyperproliferation in cancer cells by blocking the ß-catenin nuclear translocation downstream of the Wnt-regulated ß-catenin destruction complex. In normal mouse fibroblasts, loss of DKK3b disrupts cell adhesion. This newly discovered *Dkk3* gene product is an obligatory negative regulatory element in the ß-catenin signaling axis that adds a non-canonical attenuating mechanism to one of the most studied signal transduction pathways in metazoan systems. DKK3b captures ß-catenin in an extra nuclear complex with ß-TrCP preventing its nuclear translocation and serving as a gatekeeper for ß-catenin nuclear entry that modulates ß-catenin-dependent gene expression.

## Materials and methods

### Animals

Pregnant Sprague Dawley rats were purchased from Charles-River Labs. C57Bl/6J and CD1 mice were obtained from Jackson Labs and Charles River respectively. All rodents used in this study were maintained in an AALAC-accredited facility. The Animal Care and Use Committee of the University of Massachusetts Medical School (Assurance #A3306-01) approved the use of animals. All rodents were euthanized by CO_2_ asphyxiation followed by decapitation. Frozen whole brain from homozygous and heterozygous *Dkk3*^*tm1Cni*^ were the gift of Dr. C. Niehrs.

### Generation of *Dkk3*^*CFP*^ mutant mice

Zinc Finger Nuclease (ZFN) target sites within intron 2 of mouse *Dkk3* gene (NCBI: NC_000073.6) were identified (nt8312-nt8341: AGCCCCTTTTCttcacctCAGTTGTAACTG) [33, 34] and two four finger nucleases were assembled. The cDNAs encoding the zinc fingers were generated by gene synthesis (IDT) and then cloned into a pCS2 expression vector bearing the heterodimeric DD and RR versions of FokI [35]: The complete amino acid sequences of the ZFNs are *5’ HA-ZFN* (5’-GAAAAGGGGCT; DD FokI): NH_2_ YPYDVPDYATERPYKCPECGKSFSRSDTLKEHQRTHTGEKPYAC

PVESCDRRFSRSSHLTRHIRIHTGQKPFQCRICMRNFSRSDHLTQHIRTHTGEKPFACDICGRKFAQRGNLTRHTKIHTGGSQLVKSELEEKKSELRHKLKYVPHEYIELIEIARNSTQDRILEMKVMEFFMKVYGYRGKHLGGSRKPDGAIYTVGSPIDYGVIVDTKAYSGGYNLPIGQADEMQDYVEENQTRNKHINPNEWWKVYPSSVTEFKFLFVSGHFKGNYKAQLTRLNHITNCNGAVLSVEELLIGGEMIKAGTLTLEEVRRKFNNGEINF-COOH and the *3’ Flag-ZFN* (5’-CAGTTGTAACTG; DD FokI): NH_2_-DYKDDDKTERPYKCPECGKSFSRSDTLV

EHQRTHTGEKPYACPVESCDRRFSQRGNLTTHIRIHTGQKPFQCRICMRNFSRSDALRSHIRTHTGEKPFACDICGRKFARSDNLSEHTKIHTGGSQLVKSELEEKKSELRHKLKYVPHEYIELIEIARNSTQDRILEMKVMEFFMKVYGYRGKHLGGSRKPDGAIYTVGSPIDYGVIVDTKAYSGGYNLPIGQAREMQRYVEENQTRNKHINPNEWWKVYPSSVTEFKFLFVSGHFKGNYKAQLTRLNHITNCNGAVLSVEELLIGGEMIKAGTLTLEEVRRKFNNGEINF-COOH. The homologous recombination donor DNA for the mouse target *Dkk3* locus was assembled by PCR amplification. PCR primers are listed in (Table 1).

**Table 1:** Primers used in this study.

Primer	use	sequence
5HR7351F	5 HR arm	5’-AGAGAGAGAGAGACTAAGGTACTGGC
5HR38275R	5 HR arm	5’-GATCCTGAACCGGTATAACTTCGTATAATGTATGCTATACGAAG-TTATCAGTTACAACTGAGGTGAAGAAAAGG
3HR8276LAF	3 HR arm	5’-CCTGACTTAAGATAACTTCGTATAGCATACATTATACGAAGTTA-TAAAGAAACCGTTTTGTGTCTTTGATTTG
3HR9050	3 HR arm	5’-GATCCTGACTTAAGTCAAGTCATTCAGGCTGGTTG
LF	*Dkk3 locus*	5’-AAGGACCCTGCCTTGGCCACTTGG
LR	*Dkk3 locus*	5’- ACGGCATGGACGAGCTGTACC
RF	*Dkk3 locus*	5’- GCTGTACCGCTAAAGCGGCCGC
RR	*Dkk3 locus*	5’- CTCCACCCAGCTCCTGATTC
DPF1	*genotyping*	5’-TTTGGCTTGCTGGCTAAGAT
DPR2	*genotyping*	5’-GAGGGTGGTCACCAGGGT
DCF3	*genotyping*	5’-CGCCACAACATCGAGG
DCF4	*genotyping*	5’-ACCATGAGGTCTCGTCAACC
Dkk3F	Off-target PCR	5'-TGTGCCACGGCCCTTACCTT
Dkk3R	Off-target PCR	5'-GGCTAAGATAACCCTTCTGAGGTC
DlstF	Off-target PCR	5’GCAGAGGAAGTGCAAGTTTAAGGCTAGATC
DlstR	Off-target PCR	5’CAGCCATTCTAGAAACCCTGATCAAG
Rnu6F	Off-target PCR	5'-AAACTGGCTCTCGTGGGGGT
Rnu6R	Off-target PCR	5'-CACAGGTGACAGTGTGGTGG
Tcf7l2F	Off-target PCR	5'-ATGTCAGCCCACAGTGATGAGAG
Tcf7l2R	Off-target PCR	5'-CTCCCTGGAAATTGGCAGCTTG
Akap9F	Off-target PCR	5'-CCTACAGATCCGTTAAGGGTACAG
Akap9R	Off-target PCR	5'-CTCAGAGGAGGAGTCATCAAGTG
Drb1F	Off-target PCR	5’-CTCACTGTTTCTAGGAACCTTGGTTCTGTC
Drb1R	Off-target PCR	5’-GAACAGAGAAAGAAAGTCTGAGCTCAGTCC
Aldoart1F	Off-target PCR	5’-GCAGTGGCTATAGCAGAGAGGAAGAA
Aldoart1R	Off-target PCR	5’-TGCCGATGTCAAATGTAGCATGGCACTATC
Fam168bF	Off-target PCR	5'-GCATCTGTCTGCAGACCACATTTG
Fam168bR	Off-target PCR	5'-GCACGTTCACAATACTGTGGGG
Mett21eF	Off-target PCR	5’GAGAGAGAGAGAGAGAGAGAGAGAGAAGAA
Mett21eR	Off-target PCR	5’CAGAGTTAGCATTTCTTCTAGGCTCCACC
Rps6ka5F	Off-target PCR	5'-GAGGCGCATGCAGAAACTTCAC
Rps6ka5R	Off-target PCR	5'-CCTGAAAAGCCTACTAGGTCTCTC
Arhgef2F	Off-target PCR	5'-AGAGAGAGAGAGAGAGAGAGAGAG
Arhgef2R	Off-target PCR	5'-GGTGGCAAGAACCAGATGTGCT
TSS2F	CHiP	5’-AGATGGCCCCTTTTCTTCAC
TSS2R	CHiP	5’-TGGTCTCGTTGTGGTAGTTGG
EXF2	qPCR	5’-CCAGCTCTCAACTACCCTCA
EXR2	qPCR	5’-CATCAGCTCCTCCACCTCTCG
EXF3	qPCR	5’-GCTGCTAAAACGTCCTCTGAGG
EXR3	qPCR	5’-GTCTCCGTGCTGGTCTCATT
RActF	qPCR	5’-CTAAGGCCAACCGTGAAAAG
RActR	qPCR	5’-GGGGTGTTGAAGGTCTCAAA
GAPDHF	qPCR	5’-TGCCACTCAGAAGACTGTGG
GAPDHR	qPCR	5’-GGATGCAGGGATGATGTTCT
DKSF	loxP scar	5'-GAGAAGGCAGCCCCTTTTCT
DKSR	loxP scar	5'-CTTCTTCCGCCTCCATCTATCAAATC

The 824 bp 5’ homology arm (nt7415-nt8239) and 722 bp 3’homology arm (nt8342-nt9064) were appended to a loxP-TagCFP-pA-loxP cassette to create a linear 2.7 kb *Dkk3b* HR Donor DNA ZFN and HR Donor DNA. Validation was performed in the C8D1A cells (ATCC) derived from the C57Bl/6J mouse cerebellum. C8D1A cells were transfected with *Dkk3* targeted ZFN plasmids and genomic DNA was isolated 48 h later. The 681 bp target locus was PCR amplified, heat denatured, re-annealed and indel formation evaluated by Cel-1 assay [36] (Transgenomic, Inc.). HR repair of the ZFN generated DSB was validated using ssDNA oligonucleotides to insert a unique restriction site at the DSB [37]. C8D1A cells were transfected with a 96-mer ssDNA oligo with a unique EcoRI site bracketed by 45 nt long homology arms (5’CAGTCTTGGCACCTATAGAAGAGGGGAAGAGAAGGCAGCCCCTTTTGAATTCTTGTAACTGAAAGAAACCGTTTGTGTCTTTGATTTGATAGATG) along with the *Dkk3* targeted ZFN plasmids using Fugene6. The *Dkk3* target sequence was PCR amplified from gDNA isolated 48 h after transfection. HR mediated repair of the ZFN generated DSB was confirmed by the EcoRI restriction of the PCR amplified target sequence.

**Fig 1:**
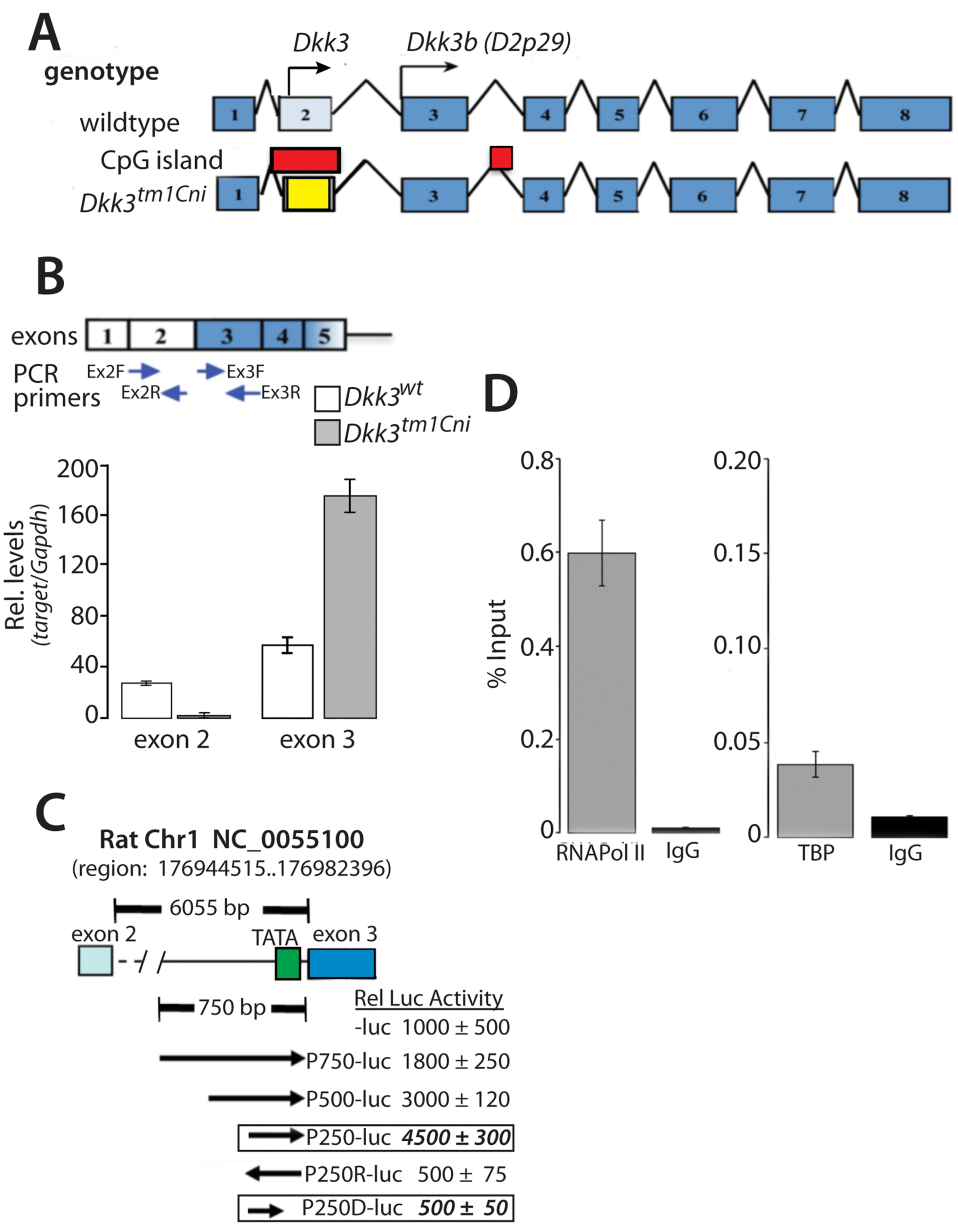
Identification of multiple transcripts originating for the *Dkk3* gene locus. (A) Schematic diagram of the *Dkk3* gene (NC_000073.6) in the wild type and *Dkk3*^*tm1Cni*^ mouse. Initiator methionine’s for *Dkk3* (NM_0154814) and *D2p29* (AF245040) indicated by arrows; CpG islands indicated by red box; LacZ-pA stop cassette in yellow. (B) Quantitative PCR of *Dkk3* containing exon 2 and exon 3 transcripts in total brain RNA from the *Dkk3*^*tm1Cni*^ mouse. Arrows indicate PCR primer sites. Data reported as means ± se of 3 individuals; each sample determined in quadruplicate. (C) Schematic diagram of rat *Dkk3* intron 2-luciferase reporter constructs. Arrows indicate orientation and location of intron 2 segments upstream of exon 3; data reported as means ± se of 3 independent experiments, each sample determined in triplicate. (D) ChIP analysis of RNA pol II and TATA box binding protein (TBP) binding to the ~66 nucleotides (nt6682-nt6948) of intron 2 adjacent to exon 3 in the rat astrocyte *Dkk3* gene; data reported as means ± se of 3 independent experiments; each sample determined in quadruplicate.

Capped, polyadenylated ZFN mRNAs were synthesized *in vitro* (Ambion), purified (Qiagen) and injected along with the 2.7 kb linear *Dkk3b* HR Donor DNA into the nucleus and cytoplasm of C57Bl/6J zygotes (UMMS Transgenic Core). Injected zygotes were implanted into the uteri of pseudo-pregnant C57Bl/6J dams and gene edited pups were identified by genotyping (see Fig 1A and Table 1 for genotyping primer pairs: DPF1:DPR2, mut-407 bp; D3F3:D3R4, mut-1031 bp; DPF1:D3R4, wt-618 bp, mut-1747bp). Insertion of the CFP cDNA at the target locus was confirmed by DNA sequencing of PCR products generated with primers LL (nt7259-nt7284) and RR (nt9058-nt9027) located 166 nt and 444 nt respectively, outside the HR region.

Predicted off-target sites were identified by PROGNOS [38]. Potential off-target sites were PCR amplified from liver gDNA (founder #19) and cloned into the pCR4 vector (Life Technologies). Eight to twelve independent clones of each potential off-target site were DNA sequenced in both directions.

The transcription initiation site in exon 2 of the mouse *Dkk3* gene was captured by 5’RACE of mRNA purified from the mutant mouse *Dkk3*^*CFP*^ cerebral cortex using the SMARTer^®^ RACE 5’/3’ kit from Clontech laboratories following manufacturer’s instructions. Briefly, mRNA was purified using the Dynabeads^®^ mRNA Direct^™^Kit from ThermoFisher according to manufacturer’s instructions. Mouse brain mRNA (500 ng) was used for 1^st^ cDNA synthesis and the 5’UTR of the *Cfp* mRNA was amplified from the 1^st^ strand cDNA by 5’RACE PCR using the kit upstream UPM primer (5’-GTAATACGACTCACTATAGGGCAAGCAGTGGTATCAACGCAGAGT) and two, independent, *Cfp*-specific downstream primers each with an appended infusion cloning sequence (bolded nucleotides): one located 384 nucleotides downstream from the *Cfp* ATG start site CFP384R (5’-**gattacgccaagctt**CTCGCCCTTCAGCTCGACGCGGTTCACC) and a second located 234 nucleotides downstream of the *Cfp* ATG start site CFP234R (5’- **gattacgccaagctt**CATGTGCTCGGGGTAGCGGGCGAAGC). Specific PCR products of ~500 bp and ~300 bp, respectively, were gel isolated, cloned into the pRACE vector (TaKaRa) and 15 clones from each *Cfp* primer were DNA sequenced.

### Cell culture and transfection

Primary astrocytes were prepared from one-day-old neonatal rat or mouse [39]. Mouse embryonic fibroblasts (MEFs) were prepared from 13.5–14.5 day old fetuses [40]. Cells were maintained at 37°C under 5% CO_2_ in Dulbecco’s modified Eagle’s medium (DMEM) containing 10% bovine calf serum (BCS), 50 units/ml penicillin, 90 units/ml streptomycin. All cell cultures were passaged every 5–7 days for up to 3 passages.

PC3, HEK293, HeLa and C8D1A cells were maintained in DMEM supplemented with 25 mM HEPES (pH 7.4) 10% fetal bovine serum, 100 units/ml penicillin, 100 μg/ml streptomycin and 2 mM L-glutamine. Cells were seeded at a density of 15,000 cells/cm^2^ 24 h prior to transfection.

Transient transfections were done with Fugene6 according to manufacturer’s instructions. Cell lysates were prepared 48 h after transfection. Each transfection experiment repeated at least 3 times and each assay point was determined in triplicate.

Tunable ectopic protein(s) expression was done using the Tet-ON expression system (Life Technologies). The Tet Repressor Protein (TR) was integrated in the genome of PC3 cells by lentivirus infection (pLenti3.3/TR, Life Technologies) and cell selected with G418. TR expressing PC3 cells were transfected with pTREX-DKK3-HA or pTREX-Flag-IBS and ectopic protein expression induced by addition of increasing concentrations of doxycycline to the growth medium.

### Luciferase reporter assays

Promoter analysis was done with PCR isolated segments of intron 2 of the rat *Dkk3* gene. The Tcf promoter [41] (M50 Super 8x TOPflash) was a gift from Randall Moon (Addgene plasmid # 12456)); the E-Cad promoter [42] was a gift from Eric Fearon (Addgene plasmid # 19290)); and the E2F promoter[43] was the gift of Jason Chen. All promoter cDNAs were cloned into the pGL4 firefly luciferase vector (Promega). Cells were transfected with the individual promoter-reporter plasmids using Fugene6. After 48 h, cell lysates were prepared and luciferase assays were done according to manufacturer’s instructions (Promega). ß-catenin signaling was evaluated in cells co-transfected with the Wnt expression plasmid, pcDNA-Wnt1 [44]; pcDNA-Wnt1 was a gift from Marian Waterman (Addgene plasmid # 35905). Each promoter-reporter experiment was performed in triplicate, and each experiment was repeated at least three times.

### Chromatin immunoprecipitation

Chromatin immunoprecipitation (ChIP) was done as described [45] with modifications. Primary rat astrocytes were cross-linked with 1% formaldehyde. Washed cell pellets were lysed on ice in hypo-osmotic lysis buffer, nuclei collected by centrifugation, and DNA sheared (average size 200–400 bp) by sonication in iso-osmotic sonication buffer. Clarified extracts were pre-cleared with A/G plus-agarose beads pre-coated with 2 μg/ml sonicated salmon sperm DNA and 1 mg/ml bovine serum albumin. Thirty *A*_260_ units of the pre-cleared nuclear lysate was then incubated with 3 μg of anti-RNA pol II, anti-TBP or normal Rabbit IgG and immune complexes isolated by A/G plus-agarose beads (Santa Cruz Biotech.). Immune complexes were eluted in 50 mM Tris, pH 8, 1 mM EDTA, 1% (v/v) SDS at 65°C for 15 min, adjusted to 200 mM NaCl, and cross-links broken by heating at 65°C for 16 h. Immune complexes were then treated with RNase I (1 unit/ml) and Proteinase K (20 μg/ml) and DNA purified (Qiagen). Immune-precipitated DNA and input DNA (10% total) were analyzed by real time PCR (MJ Research Thermal Cycler) using the SYBR Green PCR kit (Qiagen) and primers TSS2F and TSS2R (Table 1). μ

### Immunocytochemistry

Cells were seeded onto on poly-d-lysine (10 μg/ml) coated coverslips and grown for 24 h prior to transfection. All transfections were done with Fugene6 according to manufacturer’s instructions. After 48 h, cells were washed with ice cold PBS, fixed with 4% paraformaldehyde in PBS for 10 minutes and quenched with 10 mM glycine. Fixed cells were permeabilized with Triton X-100, blocked with 1% BSA in TRIS buffered saline containing 0.1% Tween 20 and then incubated with primary antibodies (0.2–1 μg/ml) for 1 h at room temperature. After washing, immune complexes were visualized with secondary antibody-Alexafluor conjugates for 1 h. Nuclei were stained with DAPI. Coverslips were mounted with Prolong^®^ Gold mountant (ThermoFisher). Tissue sections (5 μm) were prepared by the Morphology Core (UMMS) from paraffin embedded, formalin fixed organs from a *Dkk3*^*CFP/+*^ mutant mouse. Dewaxed sections were stained with anti-CFP antibody (1 μg/ml) and counter stained with hematoxylin and eosin. Antibodies used in this study were: anti-HA (ab1424), anti- ß-tubulin (ab6046), anti-DKK3 (ab186409) (Abcam), anti-FLAG (M2; F3165), anti-Myc (SAB4300320) anti-ß-catenin (04–958) (Sigma), anti-CFP (cat#632381) (TaKaRa), anti-ß-TrCP (mAB #4394), anti-GAPDH (mAB #2118) (Cell Signaling), and anti-Dkk3b [46]. Alexfluor^488^ and Alexafluor^568^ conjugated and HRP-conjugated secondary IgGs (cat#: A-11008, A-11034, A-11004, A11036, 31430, 31460) were purchased from Life Technologies; RDye^680^CW and IRDye^800^CW conjugated secondary IgGs (cat#: 925–68020, 925–68022, 925–32210, 925-32211were purchased from Li-COR Biosciences.

### RNAi knockdown

MEFs were infected with pools of lentiviral shRNAs in pGIPZ from (Dharmacon) archived by UMMS RNAi Core. Lentiviral pools of shRNAs (>1 x 10^6^ pfu/ml per shRNA target) targeting the open reading frame of mouse DKK3 and Dkk3b transcripts (exons 4 and 8) (V3LMM_518668, V3LMM_487209, V3LMM_487206, V3LMM_487207), mouse ß-catenin (exons 5 and 8) (V2LMM_16708, V3LMM_491202, V2LMM_4912040), and human ß-TrCP (V2LHS_33325, V2LHS_33330, V2LHS_33325) and a non-silencing control (RHS4348) were used. Cells were infected with lentiviral pools of shRNAs (>1 x 10^6^ pfu/ml per shRNA target), grown for 2 days and selected with puromycin. GFP positive shRNA expressing cells were used in all experiments.

### Total RNA isolation, and real time PCR

Total cellular RNA was isolated using Trizol Reagent (Life Technologies) or RNeasy (Qiagen) according to the manufacturer instructions. RT-PCR was performed with SuperScript First-Strand Synthesis System (Life Technologies) and primed with either Oligo(dT) or random hexamers. First strand cDNAs primed with either Oligo(dT) or random hexamers yielded identical results. Initially, real time PCR (MJ Research Thermal Cycler) was done with a SYBR Green PCR kit (Qiagen). All subsequent experiments were done using multiplexed TaqMan^®^ Probes and the VIIA Real Time PCR System (Applied Biosystems). TaqMan^®^ Fast Advanced Master Mix and taqMan^®^ probes for mus *Dkk3* exons 1–2, Mm01269624; mus *Dkk3* exons 3–4, Mm00443801; mus *Cyclin D1*; Mm00432359; mus c-*Myc*, Mm00487804; human *Cyclin D1*, Hs00765553; human c-*Myc*, Hs00153408; human *Axin2*, Hs00610344; human *Cox2*, Hs000153133; human *Wfdc2*, Hs00899484; human *Cdh2*, Hs00983056; human; human *Fosl1*, Hs04187685; human, *Tuba*, Hs0102675; and human *Gapdh*, Hs03929097 were purchased from Life Technologies.

### Co-immunoprecipitation binding studies

Cells expressing the epitope-tagged target proteins were lysed in 1X IP buffer (ThermoFisher Sci) with 150 mM NaCl, protease and phosphatase inhibitors. Lysates were incubated with anti-epitope antibody for 3 hrs at 4°C and immune complexes collected on ProteinA/G-agarose beads (Sigma). Immune complexes were washed five times with lysis buffer and eluted in Laemmli buffer containing 1% SDS. Cell extracts and immune complexes were separated by SDS–PAGE and immunoblot analysis done with anti-Flag, anti-Myc and anti-HA antibodies.

Native ß-catenin and ß-TrCP in HeLa cells were co-immunoprecipitated from cells treated for increasing times with TAT-Dkk3b. Anti-Dkk3b affinity DynaBeads^®^ (7.2 μg/mg resin) and control rabbit IgG affinity DynaBeads^®^ (8.4 μg/mg resin) were prepared by coupling affinity purified anti-Dkk3b IgG [46] and rabbit IgG, respectively, to Dynabeads M-270 (ThermoFisher) according to manufacturer’s instructions. HeLa cell lysates were prepared in 1X IP buffer (ThermoFisher) supplemented with 150 mM NaCl and 1X protease inhibitors. Cell lysates (200 μg protein) were incubated with 1 mg of anti-Dkk3b or Control affinity beads, in duplicate. Immune complexes were collected using a DynaMag^™^magnet. Acid eluates were then separated on 10% SDS-PAGE gels and target proteins identified by immunoblot. All native co-immunoprecipitation experiments were repeated three times.

### TAT-fusion protein synthesis

Dkk3b cDNA was cloned into the pTAT-HA plasmid [47], a gift from Steven Dowdy (Addgene plasmid # 35612). TAT-Dkk3b expression was induced with isopropyl ß-D-1-thiogalactopyranoside in BL21(DE3) cells. TAT-Dkk3b present in inclusion bodies was solubilized by denaturation in PBS containing 6 M urea, purified by affinity chromatography on Ni-NTA resin, and eluted with 150 mM imidazole in 6M Urea. Urea was rapidly removed by G10 spin column gel filtration and TAT- Dkk3b was stored at -20°C at 1 mg/ml in PBS at pH 6.0.

## Results

### The *Dkk3* gene encodes multiple transcripts

The unaltered ß-catenin signaling in the *Dkk3* knockout mouse (*Dkk3*^*tm1Cni*^) [20] led us to re-examine the biological relevance of an intracellular ~30 kDa DKK3 isoform (D2p29) that showed dynamic, microfilament dependent intracellular trafficking in rat astrocytes [46, 48]. Amino acid sequence alignment showed that the secreted DKK3 and D2p29 (designated hereafter Dkk3b) shared all but the N-terminal 71 residues that comprise the signal peptide sequence and N-glycosylation sites.

The *Dkk3*^*tm1Cni*^ mutant mouse was generated by replacement of the majority of exon 2 of the *Dkk3* gene with an in-frame LacZ-stop cassette [20]. Exon 2 encodes the N-terminal 71 amino acids responsible for directing DKK3 to the secretory vesicle, and also harbors a biologically important CpG island (Fig 1A).

The first codon in exon 3 of the *Dkk3* gene encodes the initiator methionine of Dkk3b from frogs to man, suggesting that Dkk3b is generated from a second *Dkk3b* transcript, possibly initiating within the 6 kb intron 2 (Fig 1A). Using exon specific qPCR to quantify *Dkk3* locus transcripts containing exon 2 or exon 3 (Fig 1B, S1 Fig), we found that all *Dkk3* transcripts present in mouse astrocyte mRNA had *Dkk3* exon 3 codons, while only ~60% generated a PCR product with *Dkk3* exon 2 specific PCR primers, suggesting that ~40% of the total *Dkk3* mRNA lacked the exon 2 codon and could presumably encode Dkk3b (S1 Fig). The specificity of the exon-specific qPCR of these *Dkk3* exons was validated using total RNA from *Dkk3*^*tm1Cni*^ mutant mouse brain; no *Dkk3* transcripts with exon 2 codons were detected and only *Dkk3* transcripts with exon 3 codons were found (Fig 1B).

Promoter: luciferase reporter assays were used to search for transcriptional regulatory elements located in intron 2 of the rat *Dkk3* gene. Robust promoter activity is found in the 750 nucleotides of intron 2 immediately upstream of exon 3 and progressive deletion from the 5’ end of this intron 2 fragment localized a functional promoter (TSS2) to the 250 nucleotides just upstream of the 5’ end of exon 3 (Fig 1C). The essential TATA box was located at -35 nucleotides upstream from the 5’ end of exon 3 of the *Dkk3* gene (Fig 1C, see construct P259D); in mouse and human *Dkk3* genes a TATA box is located ~90 nucleotides upstream of exon 3 and the transcriptional start site was determined to be at -92 nucleotides upstream of exon 3 by 5’RACE analysis of the *Cfp* mRNA surrogate of DKK3b (see S2 Fig). Chromatin immunoprecipitation (ChIP) of rat astrocyte gDNA revealed that the native TSS2 in the *Dkk3* gene bound RNA pol II (Fig 1D) and the TATA Box Binding Protein (TBP) (Fig 1D) indicating that the TATA box at -35nt in intron 2 (see Fig 1C) is functional.

### Dkk3b is a biologically active product of the *Dkk3* gene *in vivo*

**Fig 2:**
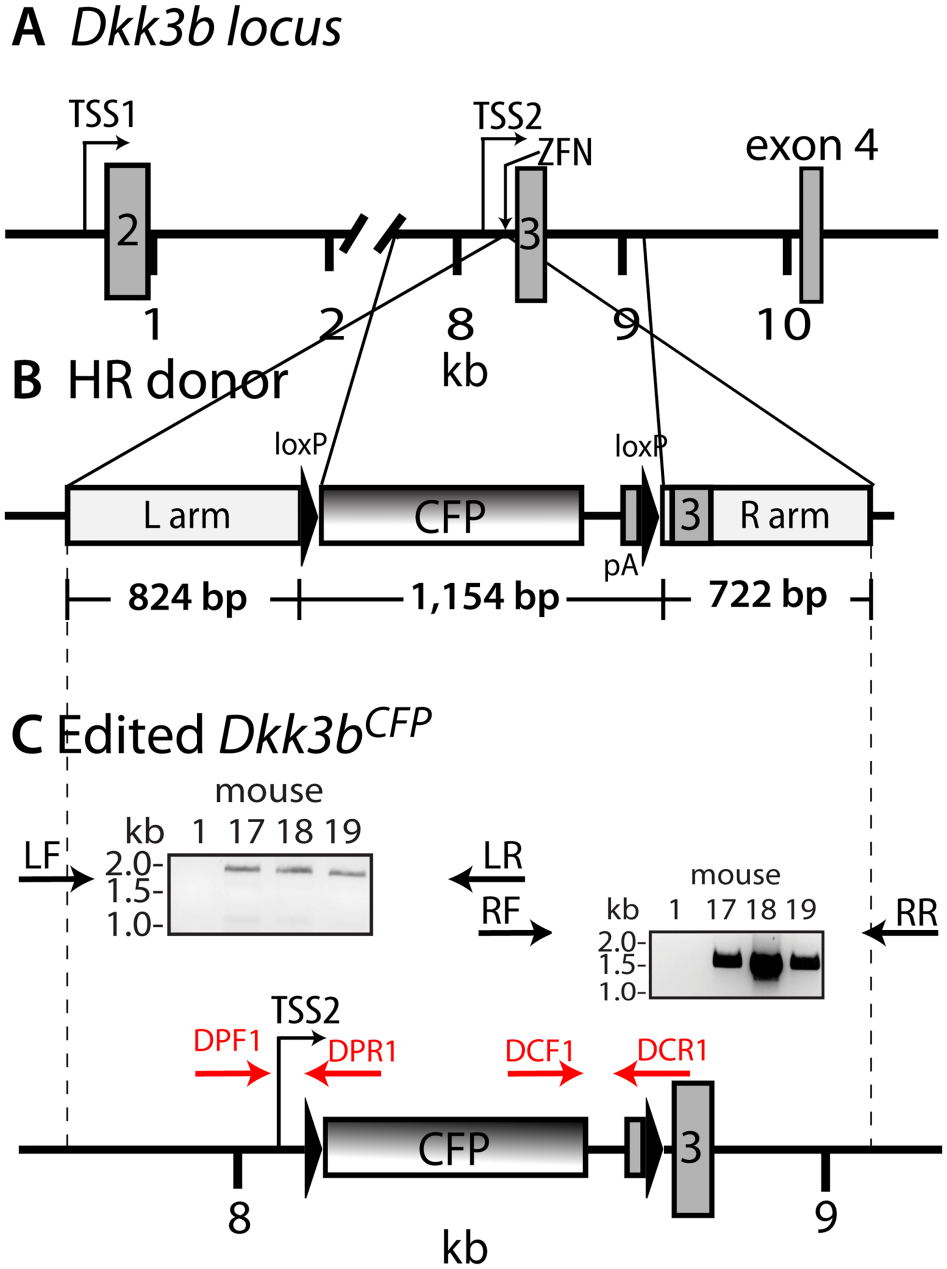
Targeting strategy for ZFN gene-editing of the *Dkk3b* locus in the mouse. (A) Organization of exons 2–4 of the wild type *Dkk3* locus. TSS1, transcriptional start site 1; TSS2, transcriptional start site 2 and ZFN targeting site. (B) Organization of the HR donor. C. Schematic diagram of the gene edited *Dkk3b*^*CFP*^ locus. Target locus modification confirmed using PCR primers anchored outside of the HR region (LF and RR) and overlapping in the CFP cds (LR and RF). PCR products (LF:LR and RF:RR) were sequenced in both directions. Genotyping PCR primers indicated by arrows (see Table 1 for sequences).

**Fig 3:**
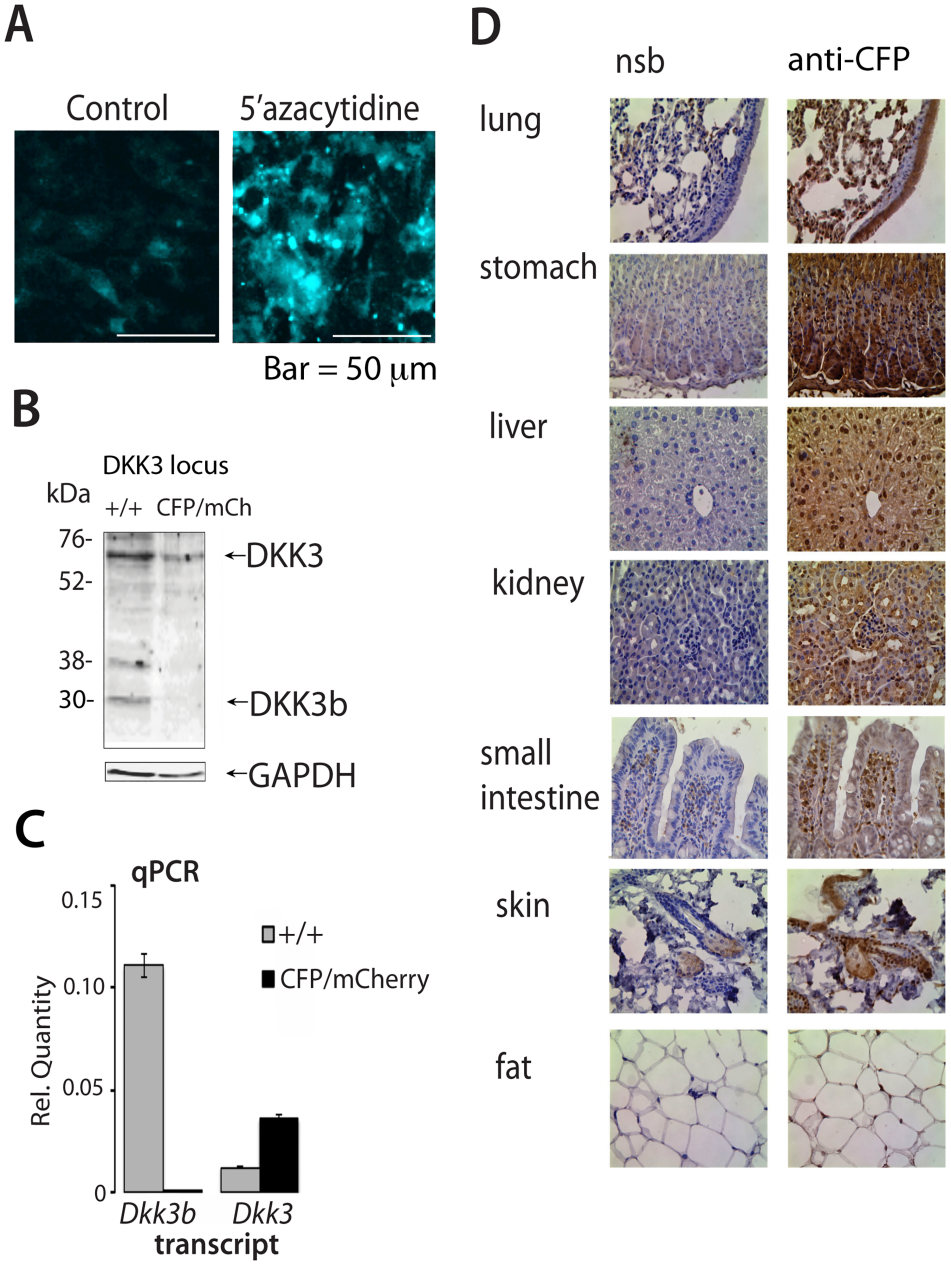
Analysis of the biological role the TSS2 driven *Dkk3b* in the ZFN gene-edited *Dkk3b*^*CFP/+*^ mouse. (A) Inhibition of DNA methyltransferase activity increases TSS2-driven CFP in *Dkk3b*^*CFP/+*^ cells. (B) DKK3 isoforms present in wild type and *Dkk3b*^*CFP/mCherry*^ MEFs (anti-DKK3 (ab186409) (Abcam). (C) QPCR analysis of *Dkk3* and *Dkk3b* transcripts in wild type and *Dkk3b*^*CFP/mCherry*^ MEFs. Data reported as means ± se, n = 3. (D) TSS2-driven, immunoreactive CFP expression in representative tissues of the *Dkk3b*^*CFP/wt*^ mouse. NSB, Normal rabbit serum, CFP, anti-CFP IgG(cat#632381) (TakaRa).

To explore the biological significance of Dkk3b, we used targeted homologous recombination (HR) driven by artificial nucleases to selectively disrupt this transcript within the mouse genome. A promoter trap knock-in strategy was devised using zinc finger nucleases [33] (ZFNs) to create a double stranded break (DSB) in intron 2 of the *Dkk3* gene between TSS2 and exon 3 that would facilitate the HR mediated insertion of a floxed CFP-stop cassette. Splice junctions at exon 3 were preserved so that TSS1-driven transcripts encoding the secreted DKK3 could produce full-length, spliced mRNA (Fig 2A–2C). The CFP insert diverts TSS2-driven transcription to the CFP surrogate resulting in the selective, functional loss of *Dkk3b* transcripts in homozygous animals. Immortal C8D1A cells, isogenic to the C57Bl/6j mouse, Cel-I assays, and single stranded oligonucleotide directed HR repair were used to validate the targeting strategy and ZFN-facilitated donor DNA HR. ZFN-generated DSBs and HR repair in the presence of the donor DNA resulted in weak expression of CFP in C8D1A cells (Fig 3A). These modified cells retain an intact exon 2 CpG island(s) upstream of the edited *Dkk3* locus [49–51]. Since CpG island methylation is common in immortalized cells [52] and presumably depresses expression of REIC (Reduced Expression in Immortal Cells)—a synonym for DKK3 [11]—we examined whether the methyltransferase inhibitor, azacytidine, would enhance expression of the TSS2-driven CFP decoy. Inhibition of DNA methyltransferase activity in the gene-edited C8D1A^cfp/wt^ cells resulted in a >5-fold increase in CFP expression (Fig 3A).

C57Bl6 mouse zygotes were then injected with ZFN^Dkk3b^ mRNAs and a linear HR donor DNA to create the *Dkk3b*^*CFP*^ knock-in mouse. Thirty-five of 65 (54%) injected one cell embryos produced viable pups, and DNA sequencing of the target locus confirmed that 1 male (#17) and 2 females (#18 & #19) (8.6%) had HR mediated insertion of a floxed CFP decoy inserted 35 nucleotides upstream from exon 3 (see Fig 2C) with intact splice junctions. No off-target mutations were found for the 10 highest predicted candidate target sites in founder #19 (Table 2). F1 progeny from crosses of the *Dkkb3*^CFP/+^ male and a wild type female showed Mendelian inheritance patterns characteristic of a single segregating allele (Table 3). The tissue distribution of *Dkk3b* expression was evaluated using the ~26 kDa CFP surrogate in the *Dkk3b*^*CFP/+*^ mutant mouse. Immunoreactive CFP was found throughout the *Dkk3b*^CFP/+^ mouse (Fig 3D) illustrating the ubiquitous nature of TSS2 activity of the *Dkk3* gene.

**Table 2:** Off-target analysis of ZFN gene edited *Dkk3b*^*CFP*^ mouse (Founder #19). Mouse C57bl6 genome GRCm38; mismatched bases are italicized and underlined.

Gene	Chr	Str	gap (nt)	location (nt)	Target sequence		# clones	
					Left nuclease	Right nuclease	wt indels	
*Dkk3*	7	-	7	112150811–12150781	5’-gaagg **CAGCCCCTTTTC** ttcacct **CAGTTGTAACTG** aaaga-3’		4	6
*Dlst*	12	+	7	85127757–85127787	5’-gcccc **CAGCCCCTT***AG***C** actggct **CAG***C***T***T***TAACTG** tatcc-3’		12	0
*Rnu6*	19	+	7	14405226–14405196	5’-aagtc **CA***A***CCCCTTTTC** tcatctc **CAGTTG***GCC***CTG** tggcg-3		10	0
*Tcf7l2*	19	+	7	55794079–55794109	5’-actga **CA***C***CCCCTTTTC** caaatga *AT***GTTGTAACTG** gcctt-3’		10	0
*Akap9*	5	+	7	3954573–3954603	5’-ccact **CA***T***CCCCTTTT***T* tctgtga **CAGTTGTAA***C***AG** agtgc-3		10	0
*Drb1*	2	+	7	76502794–76502835	5’-gtttg **CAGCCC***T***TTTT***G* tagaacc **CAGTTGTAA***AA***G** taact-3’		12	0
*Aldoart1*	4	+	7	73254288–73254328	5’-gaaat **CA***C***C***T***CCTTTTC** tttgatc **CA***T***TTG***A***AACTG** actaa-3’		10	0
*Fam168b*	1	-	6	34825844–34825873	5’-ctctc **CAGCCCC***C***TTTC** ttaatg **CAGTTGTA***A***ATG** GAGAG-3’		10	0
*Mettl21e*	1	+	6	44440591–44440619	5’-gcaaa **CAGTCCCTTTTA** tactgt **CAGGTGTACCTG** actgt-3’		8	0
*Rps6ka5*	12	+	5	100605356–100605384	5’-tcact *G***AGCC***AA***TTTTC** tggcc **CAGTTGTAACTG** tttta-3’		10	0
*Arhgef2*	3	-	5	88647980–88647952	5’-agtga **CAGCCCCTTTTC** agaaa **C***C***GT***G***GT***C***ACTG** cctgg-3’		10	0

**Table 3:** Outcome of crosses of the *Dkk3b*^*CFP/wt*^, Cre-rescued *DKK3b*^*ΔCFP/wt*^ mutant and *Dkk3b*^*wt/wt*^ mice.

	Genotype			Genotype					
Strain	male	female	pups	*Dkk3b*^*wt/wt*^		*Dkk3b*^*CFP/wt*^		*Dkk3b*^*CFP/CFP*^	
				Exp.	Obs.	Exp.	Obs.	Exp.	Obs.
C57Bl6j	*Dkk3b*^*CFP/wt*^	*Dkk3b*^*wt/wt*^	159	80	84	80	75	0	0
				(50%)	(53%)	(50%)	(47%)	(0%)	(0%)
		*Dkk3b*^*CFP/wt*^	135	33	46	68	89	33	0
				(25%)	(34%)	(50%)	(66%)	(25%)	(0%)
CD1	*Dkk3b*^*CFP/wt*^	*Dkk3b*^*wt/wt*^	159	80	84	80	75	0	0
				(50%)	(53%)	(50%)	(47%)	(0%)	(0%)
		*Dkk3b*^*CFP/wt*^	135	33	46	68	89	33	0
				(25%)	(34%)	(50%)	(66%)	(25%)	(0%)
				*Dkk3b*^*wt/wt*^		*Dkk3b*^*ΔCFP/wt*^		*Dkk3b*^*ΔCFP/CFP*^	
						*Dkk3b*^*CFP/wt*^			
CD1	*Dkk3b*^*wt/wt*^	*Dkk3b*^*ΔCFP/wt*^	56	28	27	28	29	0	0
				(50%)	(48%)	(50%)	(52%)	(0%)	(0%)
								
	*Dkk3b*^*CFP/wt*^	*Dkk3b*^*ΔCFP/wt*^	63	16	18	32	32	15	13
				(25%)	(29%)	(50%)	(51%)	(25%)	(21%)

The mRNA for the CFP surrogate of *Dkk3b* was then used to establish the TSS2-driven transcription initiation site by 5’RACE of polyadenylated mRNA isolated from the cerebral cortex of the mutant *Dkk3*^*CFP*^ mouse. The *Cfp* mRNA began at nt8284 of the *Dkk3* gene, ~90 nt upstream of the ATG start site of exon 3 of the *Dkk3* gene (S2 Fig). The 5’UTR of the *Cfp* transcript also harbored the imbedded LoxP site used to excise the promoter trap reporter illustrating that the TSS2 is functional at this mouse gene locus.

No viable homozygous mutant offspring were found after mating of heterozygous *Dkk3b*^CFP/wt^ mice (Table 3), and no homozygous mutant blastocyst implants were found as early as embryonic day 5.5 (n = 34 embryos) suggesting that DKK3b is essential for development at or near the time of embryo implantation. This outcome differs markedly from that of the *Dkk3*^*tm1Cni*^ mouse and suggests that at least one wild type *Dkk3b* allele is required for survival. The penetrance of the lethal phenotype for the single segregating *Dkk3b*^CFP^ allele was confirmed in out-crosses on the CD1 background (Table 3).

Excision of the floxed CFP cassette (ΔCFP) in the unfertilized mutant oocyte using a Sox2 promoter-driven Cre recombinase [53, 54] rescued the lethal phenotype of the *Dkk3b*^CFP^ mutation and left behind a diagnostic single 34 bp loxP remnant at the target locus. Bi-allelic, gene-edited *Dkk3b*^Δ*CFP/CFP*^ offspring were recovered from crosses of *Dkk3b*^Δ*CFP/+*^ to a *Dkk3b*^*CFP/+*^ mice (Table 3) and showed Mendelian inheritance confirming that embryonic lethality resulted from the interruption of *Dkk3b* transcription rather than any tightly linked cis gene defect(s).

Since embryonic lethality prevented the generation of homozygous mutants, e*x vivo* gene editing of heterozygous mutant MEFs was used to confirm the promoter trap knock-in strategy. Bi-allelic disruption of the *Dkk3b* locus was done in heterozygous mutant MEFs (*Dkk3b*^CFP/+^) using a second round of ZFN-initiated, HR repair to insert an mCherry reporter into the remaining wild type allele. Forty-eight hours after *ex vivo* gene editing, viable MEFs with bi-allelic mutations at the target locus expressing both fluorescent proteins were found attached to the dish, as well as free floating in the growth medium. Mutant *Dkk3b*^*CFP/mCherry*^ MEFs, both attached and free floating, were pooled and sorted by FACS. Immunoblots of sorted cell lysates showed that the *Dkk3b*^*CFP/mCherry*^ MEFs continued to express the ~65 kDa glycosylated DKK3 protein, while the 30 kDa DKK3b was absent (Fig 3B). Exon-specific qPCR confirmed preservation of the secreted *Dkk3* transcript and the selective loss of the *Dkk3b* transcript in *Dkk3b*^*CFP/mCherry*^ MEFs (Fig 3C). Attempts to propagate these mutant MEFs was prevented by the severe attachment defect associated with the DKK3b-deficient cells. These data suggest that DKK3b is necessary for cell-cell interactions, required for embryogenesis, and that TSS2-driven CFP is a surrogate for *Dkk3b* expression.

**Fig 4:**
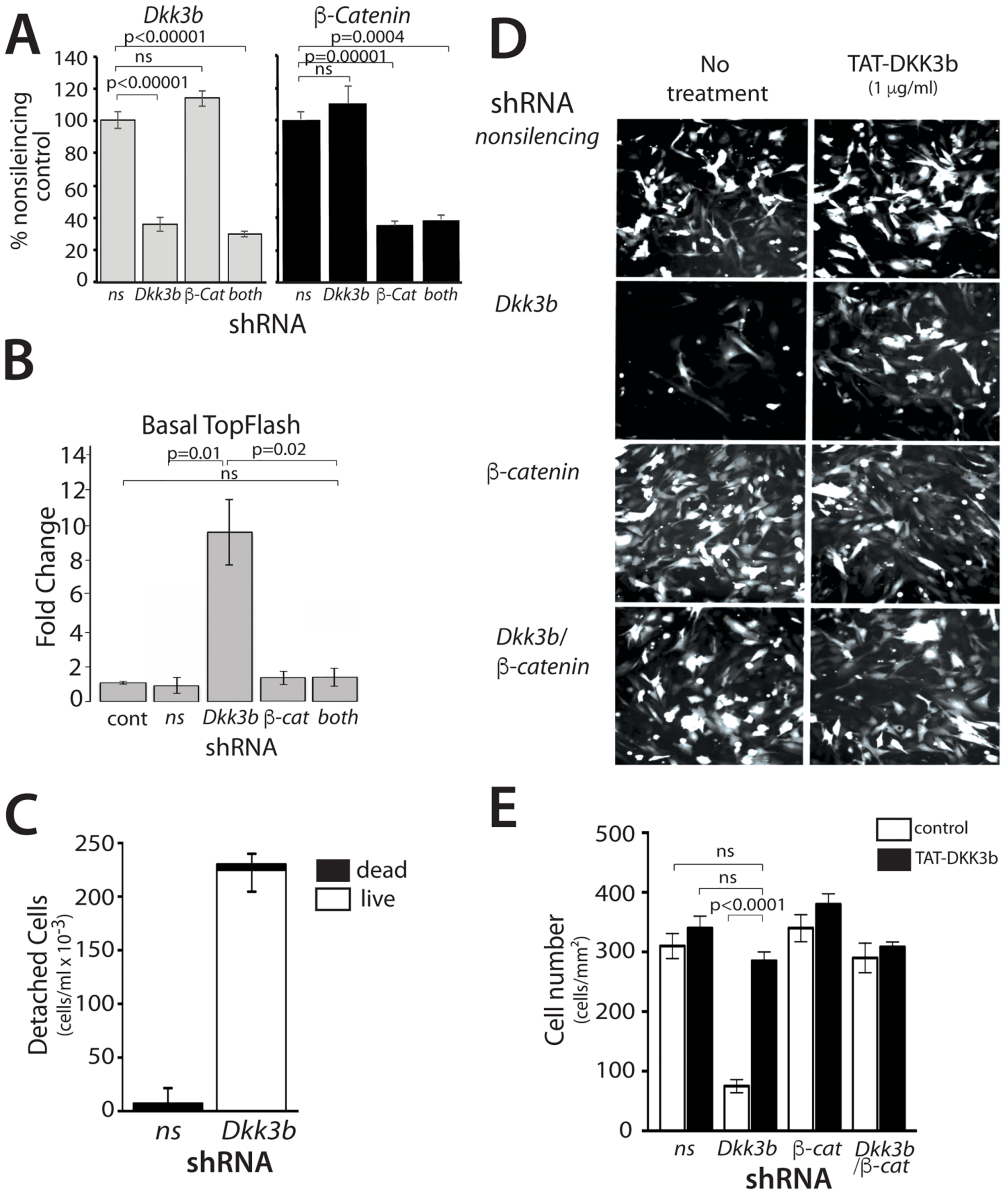
Characterization of DKK3b loss of function in MEFs. (A) QPCR analysis of shRNA knockdown of *Dkk3* and *ß-catenin* in MEFs. Data reported as means ± se, n = 3. (B) Basal TOPflash activity in shRNA KD MEFs. Data reported as the means ± se, n = 6. (C) Quantification of unattached, viable cells after 72 h in *Dkk3/Dkk3b* KD MEFs. Viability determined by Trypan blue exclusion; data reported as means ± se, n = 3. (D) Representative photomicrographs of GFP-expressing, shRNA KD MEFs. TAT-DKK3b (1 μg/ml) was added to the growth medium as indicated. GFP positive KD cells in 10 random fields were counted for each KD condition and the data reported as the means ± SD. (E) Quantitative analysis of the attached cells in part D. A total of 100 cells counted and the data expressed as cells/mm^2^. Data represent the means ± se, n = 6 wells for each condition. Taken together, these data show that our promoter-trap knock-in gene-editing strategy: i) selectively eliminated expression of the intracellular DKK3b; and ii) preserved expression of the secreted DKK3. Loss of DKK3b in MEFs led to elevated nuclear ß-catenin levels, increased ß-catenin signaling, and defective cell attachment to the substratum.

RNAi knock down was used to explore the role of DKK3b on MEF attachment, cell proliferation and its relationship to ß-catenin signaling. RNAi knockdown of the *Dkk3/Dkk3b* and/or *ß-catenin* transcripts in MEFs was confirmed by qPCR (Fig 4A). The effects of *Dkk3b* knockdown on ß-catenin dependent gene expression was examined using the ß-catenin responsive TOPflash luciferase reporter. *Dkk3b* KD MEFs showed a 10-fold increase in basal TOPflash activity compared that of the unaltered control and non-silencing KD cells (Fig 4B). Simultaneous knockdown of *Dkk3/Dkk3b* and *ß-catenin* expression completely blocked this increased signaling. Similar to the *Dkk3b*-null MEFs (see above), >70% of the viable *Dkk3/Dkk3b* KD cells were found free-floating after 3 days in culture (Fig 4C). Cell attachment and proliferation during this 3-day growth period was unaffected in cells expressing a non-silencing shRNA control or in *ß-catenin* KD cells (Fig 4D). The attachment defect in *Dkk3/Dkk3b* KD cells was completely reversed by simultaneous knockdown of *ß-catenin* mRNA (Fig 4D and 4E). Replacement of cellular DKK3b by addition of a cell penetrating TAT-DKK3b protein to the growth medium [55] completely restored cell attachment of the *Dkk3/Dkk3b* knockdown MEFs to that of the controls, but provided no additional benefit to either the *ß-catenin* KD cells or in *ß-catenin* KD:*Dkk3/Dkk3b* KD MEFs (Fig 4D and 4E).

### DKK3b modulates cell proliferation and ß-catenin signaling

**Fig 5:**
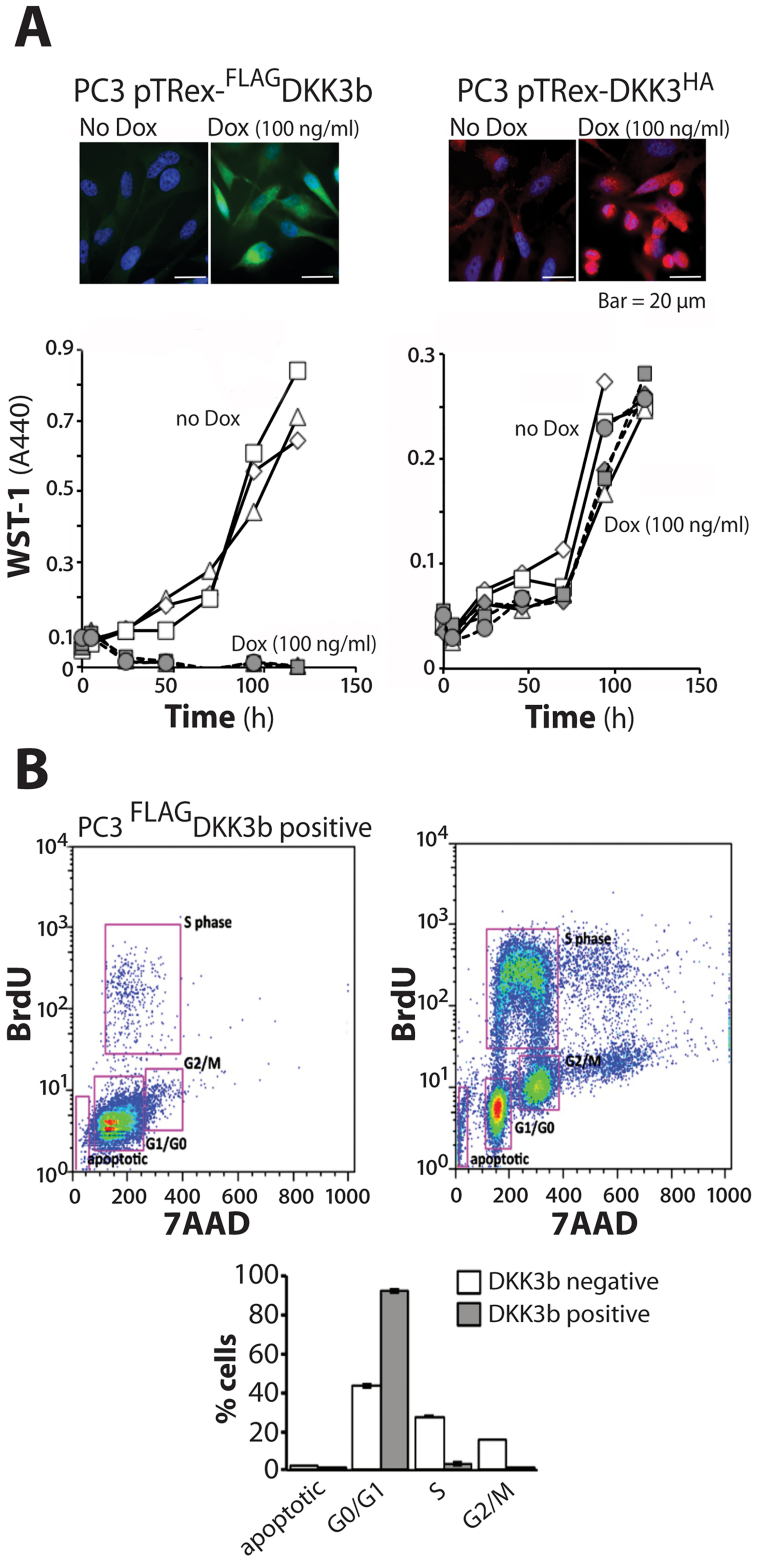
DKK3b regulation of cell proliferation. (A) Comparison of the effects of DKK3b and DKK3 on PC3 cell proliferation. Representative photomicrographs of immune-reactive, Dox-induced DKK3b and DKK3 expression in PC3 cells. Cell proliferation over 5 days (open symbols, no Dox; solid symbols, Dox; each symbol a separate experiment; time points determined in triplicate for each experiment). (B) DKK3b arrests cell proliferation at the G0/G1 phase of the cell cycle (Error bars represent the SE of three independent experiments.

The relationship between DKK3b and the ß-catenin signaling pathway was further defined by cell proliferation, promoter-driven reporter assays, and cell migration analysis in prostate and breast cancer cells. Tet-inducible constructs of the intracellular DKK3b and the secreted DKK3 were used to provide fine control over exogenous expression levels and to avoid the untoward effects of over-expression. In the DKK3/DKK3b-deficient, PC3 prostate cancer line, expression of DKK3b arrested cell proliferation of the (Fig 5A) at the G0/G1 phase of the cell cycle (Fig 5B) and led to the loss of DKK3b expressing cells by 24–36 h of induction (Fig 5A). Unlike prior over-expression studies [11, 19, 56, 57], induction of equivalent levels of secreted DKK3 did not alter PC3 cell proliferation (Fig 5A and 5B).

These data show that DKK3b has the anti-proliferative activity in cancer cells that were previously associated with gene product(s) from the *Dkk3* locus [11, 56].

**Fig 6:**
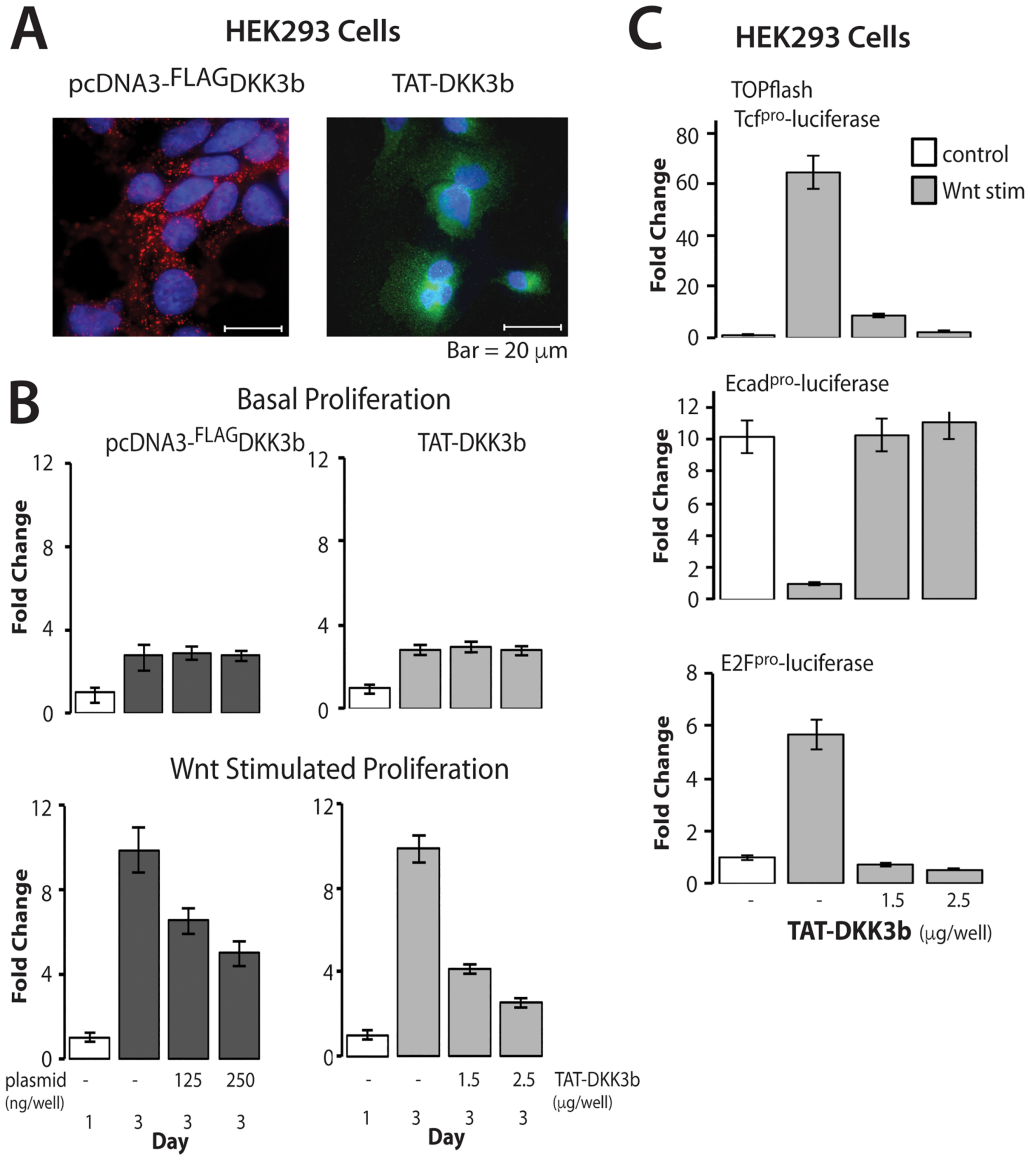
DKK3b effects on Wnt1-stimulated ß-catenin signaling. (A) Representative photomicrographs of the cellular distribution of transiently transfected DKK3b and TAT-DKK3b in HEK293 cells. DKK3b identified with anti-Flag IgG and anti-DKK3 IgG, respectively. (B) DKK3b blocks Wnt1-stimulated cell proliferation without altering basal cell proliferation. Data reported as means ± se of 4 independent experiments; n = 3 in individual experiments. Open bar—day 1; shared bars—day 3. (C) TAT-DKK3b antagonizes Wnt1-stimulated TOPflash activity and secondary ß-catenin dependent gene expression. Data reported as means ± se of 3 independent experiments; triplicate determinations done in individual experiments.

The relationship between DKK3b and ß-catenin signaling was defined by cell proliferation and promoter-driven reporter assays in HEK293 cells with reduced endogenous DKK3b. Basal cell proliferation was unaffected by ectopic DKK3b produced either by transient transfection and by addition of the TAT-DKK3b (Fig 6A and 6B). On the other hand, Wnt-stimulated cell proliferation was progressively slowed, but not arrested in cells treated with DKK3 by either transient transfection or by TAT-Dkk3b (Fig 6B). The more robust decrease in proliferation observed with TAT-DKK3b treatment is likely due to the more uniform delivery of the tumor suppressor to the cell monolayer. Concentrations of TAT-DKK3b ≥2.5 μg/ml completely silenced Wnt-stimulated cell proliferation without altering basal cell proliferation (Fig 6B, compare basal and Wnt stimulated proliferation at 2.5 μg/ml TAT-DKK3b).

Primary and downstream promoter-luciferase reporter assays determined the effects of DKK3b on ß-catenin-driven gene expression. HEK293 cells were co-transfected with Wnt1 and promoter-driven firefly luciferase constructs paired with a control CMV-driven renilla luciferase cDNA, and then treated with TAT-DKK3b for 24 h. Wnt1 stimulated cells showed a 65-fold increase in TOPflash activity and TAT-DKK3b completely arrested expression of this canonical ß-catenin reporter (Fig 6C). DKK3b also modulated downstream ß-catenin regulated pathways that reduce cell adhesion (ECad promoter [58]) and promote cell cycle progression (E2F promoter [59]). Wnt1 silenced E-Cad promoter activity by 90%, but the presence of TAT-DKK3b restored promoter activity to basal levels (Fig 6C). Similarly, Wnt1 increased E2F-promoter activity 6-fold, but the presence of TAT-DKK3B maintained E2F-promoter activity at baseline levels (Fig 6C). Taken together, these data show that DKK3b modulates multiple aspects of ß-catenin signaling.

### DKK3b blocks nuclear translocation of ß-catenin

Yeast two hybrid screens showed that DKK3 interacted with the E3 ubiquitin protein ligase, ß-TrCP, and that this complex captured cytoplasmic dephosphorylated ß-catenin [17], although the source of the essential intracellular effector produced by the *Dkk3* gene was not identified [17, 18]. DKK3b provides just such an intracellular effector.

**Fig 7:**
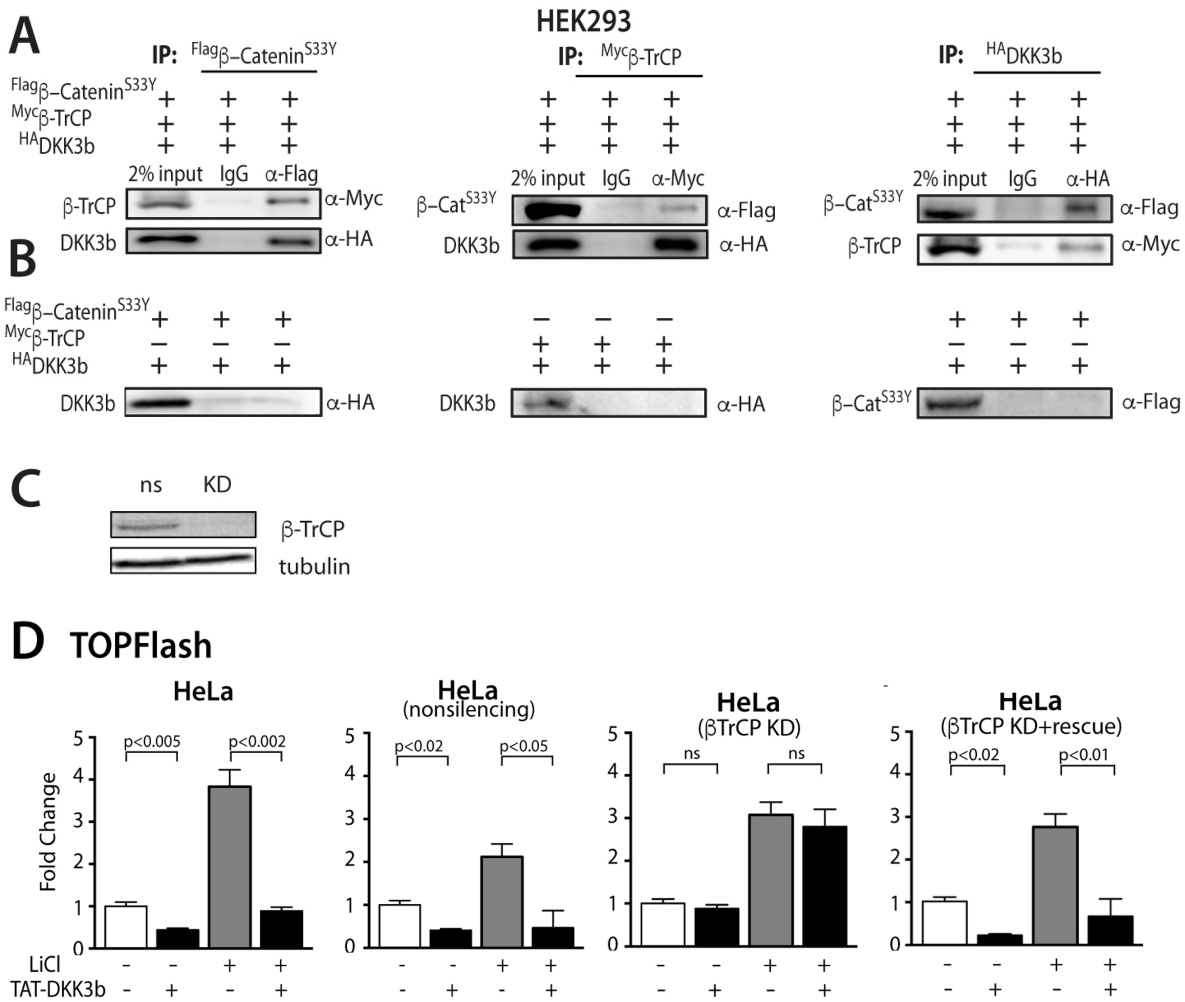
Characterization of the DKK3b:ß-TrCP:ß-catenin complex and its effects of ß-catenin nuclear translocation/signaling. (A) Co-IP of DKK3b, ß-TrCP and ß-^S33Y^catenin from HEK293 cell lysates. Epitope tagged targets were expressed by transient transfection in HEK293 cells, immune precipitates collected by Protein A/G Sepharose, and co-precipitating partners were analyzed by immunoblot with epitope specific antibodies. (B) Co-IP of HEK293 cell lysates lacking one binding partner. (C) shRNA knockdown of ß-TrCP in HeLa cells. Immunoblots done with anti-TrCP IgG. (D) Effects of *ß-TrCP* KD and ß-TrCP rescue on TAT-DKK3b dependent inhibition of TOPflash activity in HeLa cells. Unaltered, non-silencing control, *ß-TrCP* KD cells, and rescued *ß-TrCP* KD cells expressing a mouse ß-TrCP rescue plasmid were stimulated with ±LiCl for 16h in the absence or presence of TAT-DKK3b (5 μg/ml). TOPflash activity reported as fold change from resting HeLa cells. Data are reported as the means ± se (n = 4); each experiment was repeated 3 times.

Co-IP studies were done with lysates of HEK293 cells constitutively expressing epitope-tagged DKK3b, ß-TrCP and the constitutively active S33Y mutant of ß-catenin that evades the destruction complex. When all three proteins were present, immune precipitates of Flag-^S33Y^ß-catenin also contained both ß-TrCP and DKK3b, while control IgG precipitates failed to capture any epitope tagged targets (Fig 7A). Similarly, immune precipitates of myc-ß-TrCP contained both ^S33Y^ß-catenin and DKK3b; immune precipitates of HA-DKK3b contained both ^S33Y^ß-catenin, and ß-TrCP (Fig 7A). Co-IP studies of cell lysates expressing only two of the three binding partners (Myc-ß-TrCP and HA-DKK3b) or (FLAG-^S33Y^ß-catenin and HA-DKK3b) failed to show any interaction between these epitope-tagged binding partners (Fig 7B). These data suggest that DKK3b captures unphosphorylated ß-catenin in a complex with ß-TrCP and that all three partners are required to assemble this complex.

The role of ß-TrCP in the DKK3b:ß-TrCP:ß-catenin complexes on nuclear trafficking and gene expression was examined in *ß-TrCP* KD HeLa cells. Immunoblot analysis confirmed shRNA dependent loss of the *ß-TrCP* transcript in KD cells (Fig 7C). To maximize nuclear localization of ß-catenin, control and *ß-TrCP* KD cells were treated with LiCl, a chemical mimic of Wnt that stabilizes cytosolic ß-catenin by inhibiting its phosphorylation by GSK-3 in the destruction complex [60]. The effects of the DKK3b:ß-TrCP:ß-catenin complex on ß-catenin-driven gene expression in *ß-TrCP* KD HeLa cells was then evaluated using the TOPflash assay. In both resting and LiCl-stimulated control and non-silencing KD cells, addition of TAT-DKK3b silenced TOPflash activity (Fig 7D). In *ß-TrCP* KD cells, TAT-DKK3b had no effect on TOPflash activity, whereas rescue of the *ß-TrCP* KD by transfection with mouse ß-TrCP cDNA restored the ability of TAT-DKK3b to arrest TOPflash activity in both resting and LiCl-stimulated cells. These data show that the DKK3b:ß-TrCP:ß-catenin complex alters the ability of the ß-catenin to enter the cell nucleus and blocks ß-catenin-driven gene expression.

**Fig 8:**
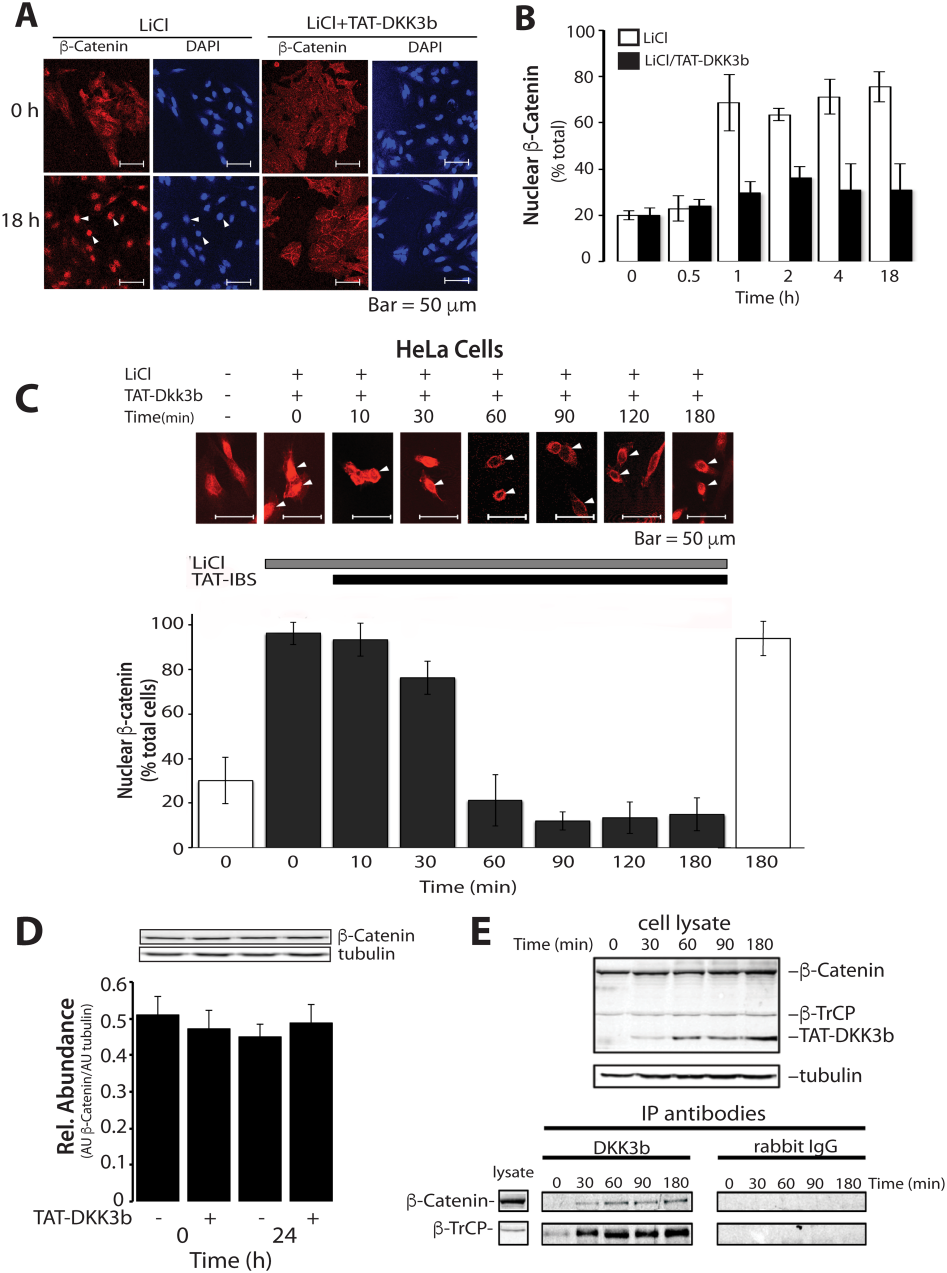
Effects of TAT-DKK3b on the dynamics of ß-catenin nuclear translocation. (A) Representative photomicrographs of the distribution of ß-catenin in control (unstimulated) and LiCl-stimulated HeLa cells ± TAT-DKK3b (arrows—cell nucleus). (B) Time course of LiCl-induced nuclear translocation of ß-catenin in HeLa ± TAT-DKK3b (5 μg/ml). At least 200 cells were counted in 20 random fields from 3 individual slides; data reported as means ± SD. (C) Time course of the effects of TAT-DKK3b on steady-state nuclear ß-catenin in LiCl-stimulated HeLa cells. Nuclear ß-catenin positive cells were scored as in B, and the data reported as means ± SD of 3 independent experiments. (D) Immunoblot of the effects of TAT-DKK3b on ß-catenin levels in HeLa cell lysates. Data reported as means ± se, n = 3. (E) Time dependent assembly of the TAT-DKK3b:ß-TrCP:ß-catenin complex in LiCl-stimulated HeLa cells. Cell lysates were incubated with anti-DKK3b-IgG or NRS-conjugated DynaBeads, and co-precipitating proteins determined by immunoblot with anti-target antibodies.

The effects of DKK3b on the dynamics ß-catenin nuclear trafficking was determined in LiCl stimulated HeLa cells. HeLa cells lack native *Dkk3/Dkk3b* expression and show accelerated ß-catenin-driven cell proliferation [61]. As expected, HeLa cell ß-catenin was distributed throughout the cell interior with marginal localization at the cell periphery (Fig 8A). In the absence of DKK3b, LiCl led to the rapid accumulation of ß-catenin in the nucleus reaching maximal levels after 60 min in >70% of the treated cells, and this remained constant for 18 h when LiCl was present (Fig 8A and 8B). Addition of TAT-DKK3b along with LiCl completely blocked the accumulation of nuclear ß-catenin for up to 18 h.

The time dependent efflux of nuclear ß-catenin due to TAT-DKK3b was determined in HeLa cells stimulated with LiCl for 18 hrs. Addition of TAT-DKK3b to these maximally stimulated cells resulted in the rapid loss of nuclear ß-catenin, beginning within 30 min and reaching maximal suppression by 60 min (Fig 8C). TAT-DKK3b-suppressed nuclear ß-catenin below levels in untreated HeLa cells for at least 3 h. Total HeLa cell ß-catenin levels were unaffected by TAT-DKK3b (Fig 8D) indicating that the loss of ß-catenin from the nucleus in TAT-DKK3b treated cells was due to cellular redistribution rather than enhanced ß-catenin degradation. Anti-DKK3b-IgG-affinity beads were used to capture DKK3b associated native proteins in the LiCl-stimulated HeLa cell lysates (Fig 8E). The loss of nuclear ß-catenin in LiCl-stimulated cells was accompanied by the time-dependent accumulation of both ß-catenin and ß-TrCP in a complex with the TAT-DKK3b (Fig 8E). Total cellular levels of both ß-catenin and ß-TrCP were unaltered during the 3 h TAT-DKK3b treatment period, while intracellular DKK3b showed a time-dependent accumulation in the complex that paralleled the loss of ß-catenin from the cell nucleus (Fig 8E). Thus, the DKK3b:ß-TrCP:ß-catenin inhibitory complex is formed rapidly, interrupts the dynamics of nuclear import/export, and defines the molecular basis for the silencing of ß-catenin signaling by DKK3b.

Since DKK3b silenced both TOPflash activity and captured the native transfactor in an inhibitory complex with DKK3b:ß-TrCP, we determined the effects of TAT-DKK3b on native gene expression in resting and LiCl-stimulated HeLa cells. Like the TOPflash assay, addition of TAT-DKK3b to unstimulated HeLa cells led to a 40–60% decrease in both native *CyclinD1* and *c-Myc* transcripts after 16 h (S3A Fig). Similarly, in both resting and LiCl-stimulated cells, the expression of 5 representative ß-catenin dependent genes *Cdh2*, *Axin2*, *Cox2*, *Wfdc2 and Fosl1* all showed DKK3b dependent silencing (S3B Fig), while expression of the ß-catenin independent *Gapdh* used for normalizaiton, and alpha tubulin (*Tuba)* transcripts were unaffected. These data show that both reporter and intrinsic gene expression are modulated by ability of DKK3b to control ß-catenin translocation to the cell nucleus, and that this action depends on the the ability of DKK3b to capture the native transactivator in an extranuclear complex with ß-TrCP.

## Discussion

DKK3 is the enigmatic member of an ancient family of secreted glycoproteins that regulate the Wnt/ß-catenin pathway by interrupting the assembly of a functional Wnt liganded receptor [4, 11]. It is the only unambiguous tumor suppressor in the family, and a diverse literature links DKK3 and tumor suppression to the ß-catenin pathway [11]. Unfortunately, the inability of DKK3 to block Wnt receptor assembly due to steric hindrance [4, 12] poses a significant challenge to our understanding of its tumor suppressor function. Yeast two-hybrid screens [17] showed that DKK3 formed a complex with ß-TrCP, and dephosphorylated ß-catenin that prevented transfactor nuclear translocation [17], but Lee and co-workers did not identify the authentic intracellular DKK3 isoform responsible for this biology.

The recognition that the *Dkk3* gene encodes a second gene product, DKK3b—a vital intracellular protein that regulates ß-catenin trafficking—provides the missing component that connects the *Dkk3* gene to its regulation of the ß-catenin signaling pathway. DKK3b acts on the ß-catenin signaling pathway independent of the Wnt modulated destruction complex and modulates a diverse array of cellular functions, such as cell proliferation, cell attachment, embryogenesis and gene expression. Positioned downstream of the Wnt regulated degradation complex, DKK3b modulates ß-catenin trafficking to the nucleus. In astrocytes, DKK3b rapidly shuttles between the perinuclear space and the plasma membrane using myosin motors and actin fibers [48, 62], suggesting that it may carry ß-catenin back the cell periphery for reuse in the adherens complex remodeling. The ability of DKK3b to repair the attachment defect observed in DKK3b-null MEFs suggests that DKK3b plays a key role in the delivery of ß-catenin to its plasma membrane reservoir.

The *Dkk3* gene joins a growing list of mammalian genes that use multiple alternate promoters to achieve functional diversity [63, 64]. Our identification of a second TSS element in the *Dkk3* gene and the demonstration that it generates a second transcript encoding a vital intracellular isoform was unexpected. Using targeted gene editing to abolish *Dkk3b* expression, while preserving expression of its sister (*Dkk3*), we found that DKK3b was essential for early embryonic development at or near implantation, dramatically different from the *Dkk3*^*tm1Cni*^ mutant mouse where secreted *Dkk3* expression was inactivated [20]. Despite the presence of the entire Wnt/ß-catenin pathway from oocyte to the late blastocyst stage [65, 66], active canonical Wnt signaling is not observed in pre-implantation blastocysts [67], and is first observed post-implantation at E6.5 [68]. Using a Wnt reporter mouse expressing a functional mutant of ß-catenin that evades the destruction complex, Kemler et al [69] found the stabilized ß-catenin in the cytoplasm of pre-implantation embryos, but that it did not traffic to the nucleus and no Wnt reporter expression was observed. They suggested that cellular mechanism(s) other than the canonical destruction complex [69] were responsible for keeping the stabilized ß-catenin out of the cell nucleus before implantation. Since DKK3b is an important gatekeeper of ß-catenin nuclear translocation, its loss in the zygote is likely to unleash ß-catenin signaling that has detrimental consequences on the developing embryo both before implantation and at this critical developmental event.

DKK3b-deficient MEFs showed both elevated ß-catenin signaling and cell attachment defect(s); two events likely to interrupt orderly development of the embryo. These disruptions in the signaling pathway were rescued by replacement with ectopic DKK3b or by simultaneous knockdown of ß-catenin. These data illustrate that the loss of DKK3b directly leads to aberrant ß-catenin signaling and disrupts cell-cell and cell-substrate interactions.

Unlike its ubiquitous expression in somatic cells, silencing of the *Dkk3* gene is common in cancer and ectopic over-expression of DKK3 arrests tumor cell growth [16–19]. Analysis of intracellular product of the *Dkk3* gene, revealed that DKK3b arrests Wnt stimulated cell proliferation and selectively silences ß-catenin dependent gene expression in both immortal and cancer cells. The ability a purified cell-penetrating DKK3b protein to fully restore control of ß-catenin signaling by partnering with ß-TrCP to capture the transfactor in an extra nuclear complex offers a promising new therapeutic target for ß-catenin driven hyper-proliferative disease(s) like cancer.

**Fig 9:**
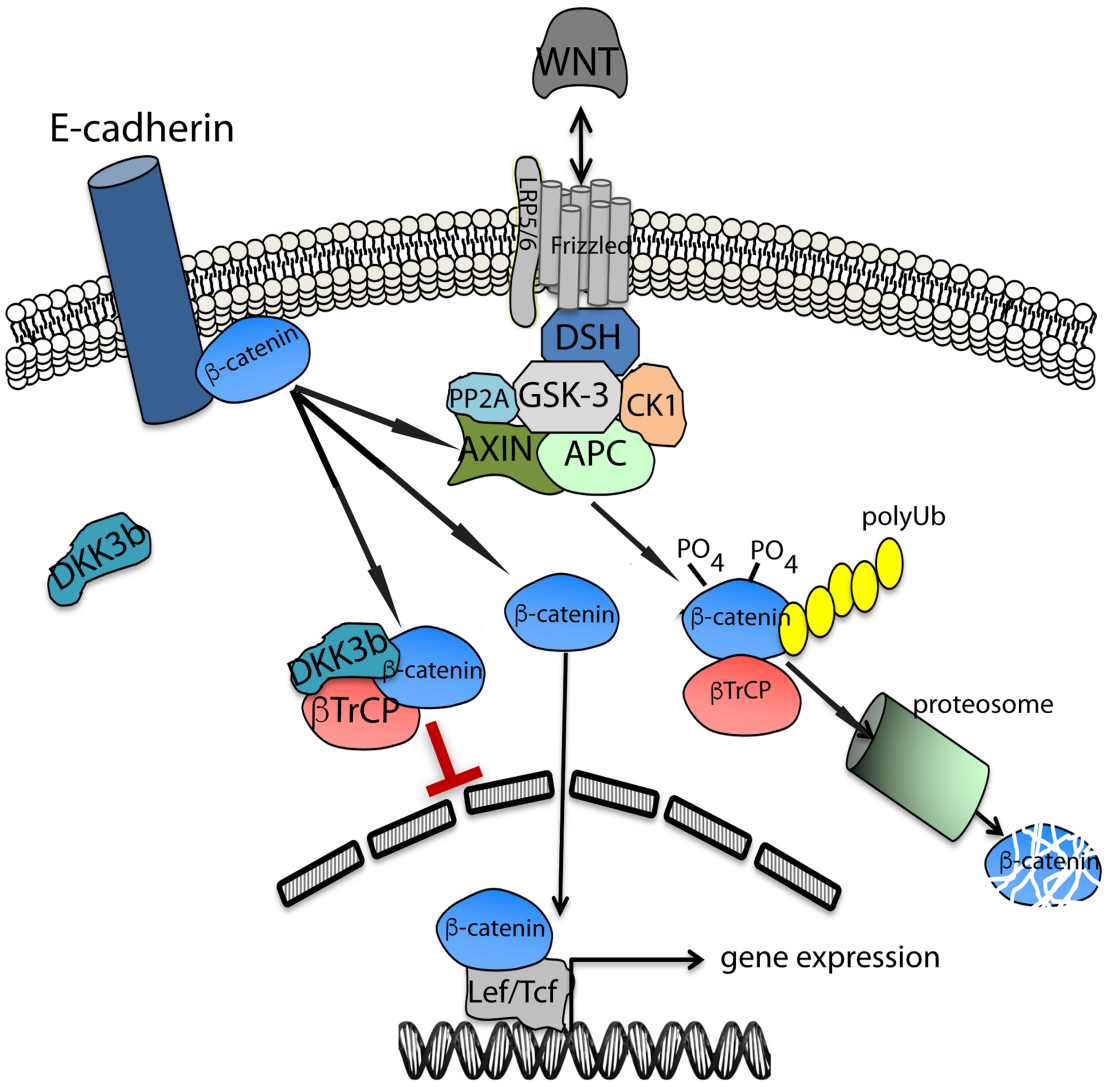
Schematic diagram of the novel regulatory role of DKK3b in the Wnt/ß-catenin signaling pathway. DSH, Disheveled; GSK-3, Glycogen synthase kinase 3 beta; CK1, Casein kinase 1; PP2A, Protein Phosphatase 2A; APC, Adenomatous Polyposis Coli; ß-TrCP, ß-Transducin Repeat-Containing Protein; Ub, ubiquitin.

An essential partner in the molecular mechanism of DKK3b action is ß-TrCP, an F-box protein with WD40 repeats that captures a broad range of protein targets through a consensus dephosphorylated 6 amino acid long degron domain in the target protein(s) that is located 10 to 20 residues downstream of the lysine used for ubiquitin conjugation [70]. DKK3b captured dephosphorylated ß-catenin in a complex with ß-TrCP, prolonged the biological half-life of the transfactor, and prevented its translocation to the nucleus (Fig 9). Direct analysis of the dynamics of ß-catenin nuclear entry and exit showed that the tripartite complex trapped the transfactor outside the nucleus providing a clear molecular mechanism for the DKK3b-dependent regulation of this pathway. Since the N-terminal localized degron of ß-catenin docks with ß-TrCP, the ability of DKK3b stabilize transient interaction(s) between the dephos-degron of ß-catenin and ß-TrCP during “kiss-and-run” interaction(s) [71], suggests that other ß-TrCP ligands that similarly engage this E3 ligase are potential regulatory targets. These include members of most of the kinase signaling cascades that impact cell growth, motility, and apoptosis [71–74]. Further studies are required to determine if DKK3b captures other ß-TrCP interacting proteins and impacts their signaling pathways.

DKK3b is a novel and essential component of the Wnt/ß-catenin signaling pathway that plays a key role in both development and cancer. It serves as a gatekeeper for ß-catenin nuclear entry, directly modulates this pro-proliferative signaling molecule, and provides an important new point of control that impacts the regulatory pathways responsible for differentiation, lineage specification, pluripotency and oncogenesis.

## Supporting information

S1 Fig Exon specific qPCR analysis of *Dkk3* transcripts in mouse astrocytes. (A) The position of the PCR primer pairs used for amplification of exon 2 and exon 3 shown on the *Dkk3* cds. (B) Validation of the *Dkk3* exon 2 and exon 3 primer sets using increasing concentrations of 1^st^ strand cDNA primed total RNA isolated from two independent mouse astrocyte preparations. Each data point determined in triplicate. (C) *Dkk3* mRNA levels were normalized to GAPDH mRNA. Data shown as (mean ± SE) from 3 independent experiments.
(TIF)

S2 Fig 5’RACE analysis of *Cfp* mRNA from the *Dkk3b*^*CFP*^ mouse. (A) Map of the insertion of the CFP promoter trap in gene-edited intron 2 of the *Dkk3* gene. TSS2 is positioned upstream of the forward LoxP site (black box) of the gene and the downstream LoxP site (in black) is positions 35 nt upstream of exon 3. Position of the CFP234 5’RACE primer indicated by arrow. (B) Sequence of the 5’UTR of the Cfp mRNA captured by 5’RACE highlighted in yellow.
(TIF)

S3 Fig Effects of TAT-DKK3b on basal and LiCl-stimulated gene expression in HeLa cells. (A) Basal *CyclinD1 and Myc* expression in HeLa cells ±TAT-DKK3b for 16 h. (B) Native ß-catenin dependent gene expression in HeLa cells. Cells were stimulated with ±LiCl in the absence or presence of TAT-DKK3b for 16 h. QPCR data are reported as % of unstimulated controls for each target transcript and expressed as means ± se, n = 9. Gene products probed: *Cdh2*, N-cadherin; *Axin2*, Axin2; *Cox2*, cyclooxygenase-2; *Wfdc2*, *WAP* Four-disulfide Core Domain 2; *Fosl1*, FOS Like Antigen 1; *Tuba*, alpha tubulin.
(TIF)
