# Correction: The *Dkk3* gene encodes a vital intracellular regulator of cell proliferation

**DOI:** 10.1371/journal.pone.0184458

**Published:** 2017-09-01

**Authors:** Jack L. Leonard, Deborah M. Leonard, Scot A. Wolfe, Jilin Liu, Jaime Rivera, Michelle Yang, Ryan T. Leonard, Jacob P. S. Johnson, Prashant Kumar, Kate L. Liebmann, Amanda A. Tutto, Zhongming Mou, Karl J. Simin

The images for S1 and S2 Figs are incorrectly switched. The image that appears as S1 Fig should be S2 Fig, and the image that appears as S2 Fig should be S1 Fig. The figure captions appear in the correct order. Please see the correct S1 and S2 Figs here.

## Supporting information

S1 Fig Exon specific qPCR analysis of *Dkk3* transcripts in mouse astrocytes. (A) The position of the PCR primer pairs used for amplification of exon 2 and exon 3 shown on the *Dkk3* cds. (B) Validation of the *Dkk3* exon 2 and exon 3 primer sets using increasing concentrations of 1^st^ strand cDNA primed total RNA isolated from two independent mouse astrocyte preparations. Each data point determined in triplicate. (C) *Dkk3* mRNA levels were normalized to GAPDH mRNA. Data shown as (mean ± SE) from 3 independent experiments.
(TIF)

S2 Fig 5’RACE analysis of *Cfp* mRNA from the *Dkk3b*^*CFP*^ mouse. (A) Map of the insertion of the CFP promoter trap in gene-edited intron 2 of the *Dkk3* gene. TSS2 is positioned upstream of the forward LoxP site (black box) of the gene and the downstream LoxP site (in black) is positions 35 nt upstream of exon 3. Position of the CFP234 5’RACE primer indicated by arrow. (B) Sequence of the 5’UTR of the Cfp mRNA captured by 5’RACE highlighted in yellow.
(TIF)
