# Supplementary figures and images for: Correction: The Dkk3 gene encodes a vital intracellular regulator of cell proliferation
Source: PLoS One. 2017 Sep 1;12(9):e0184458. doi: 10.1371/journal.pone.0184458 (PMC5580920; doi:10.1371/journal.pone.0184458)

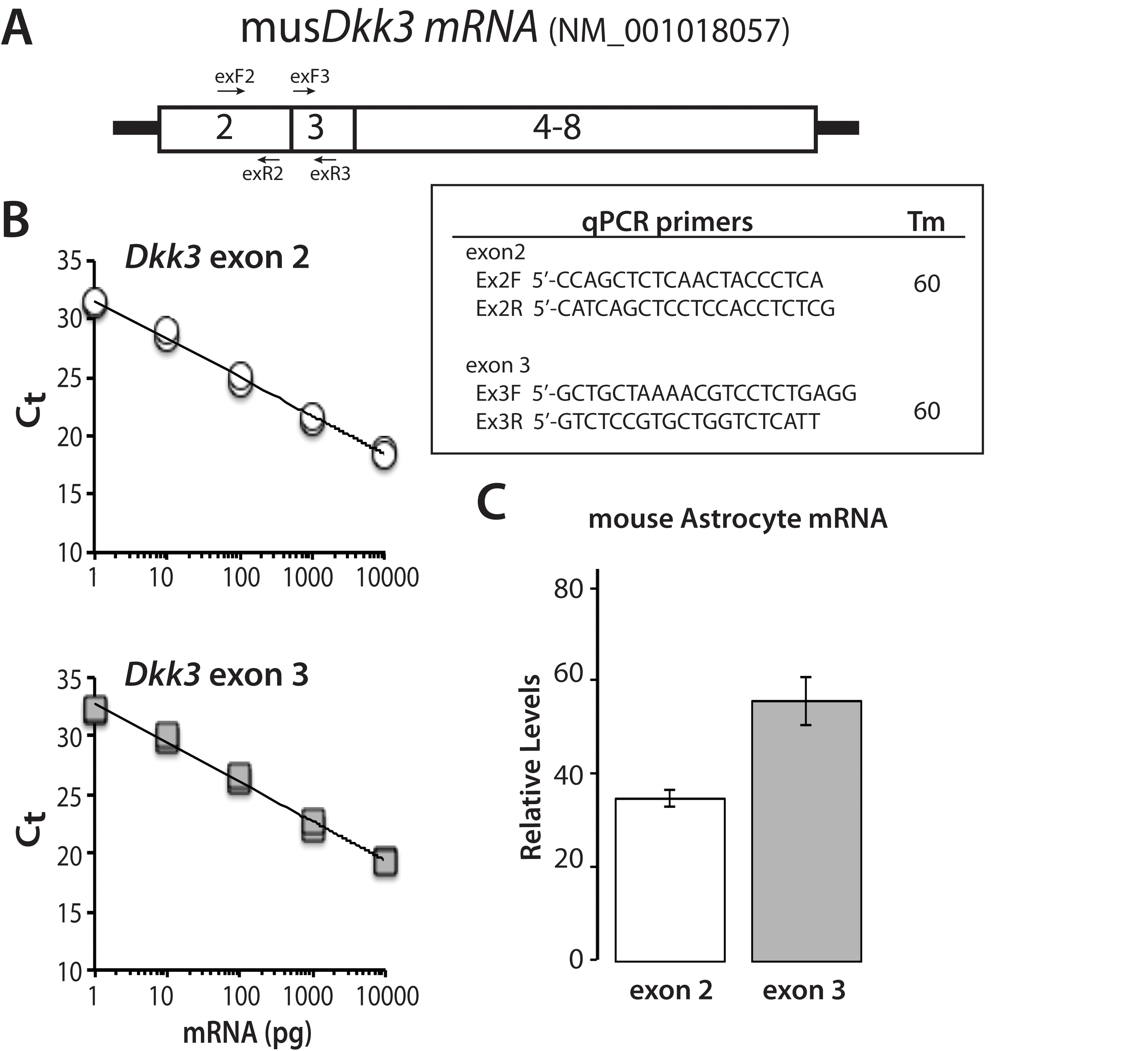

Supplement: S1 Fig — (A) The position of the PCR primer pairs used for amplification of exon 2 and exon 3 shown on the Dkk3 cds. (B) Validation of the Dkk3 exon 2 and exon 3 primer sets using increasing concentrations of 1st strand cDNA primed total RNA isolated from two independent mouse astrocyte preparations. Each data point determined in triplicate. (C) Dkk3 mRNA levels were normalized to GAPDH mRNA. Data shown as (mean ± SE) from 3 independent experiments. (TIF) [file pone.0184458.s001.tif]

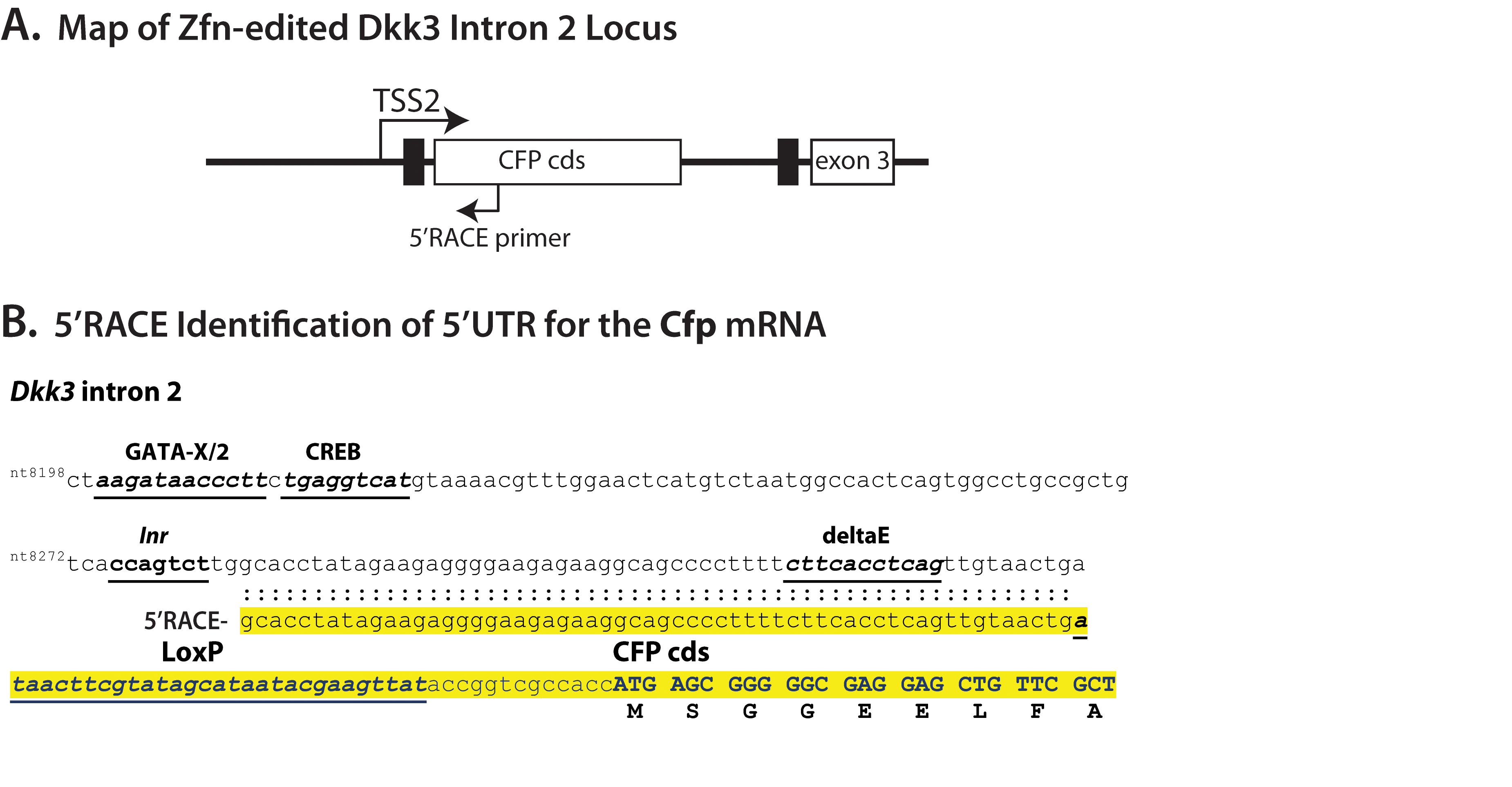

Supplement: S2 Fig — (A) Map of the insertion of the CFP promoter trap in gene-edited intron 2 of the Dkk3 gene. TSS2 is positioned upstream of the forward LoxP site (black box) of the gene and the downstream LoxP site (in black) is positions 35 nt upstream of exon 3. Position of the CFP234 5’RACE primer indicated by arrow. (B) Sequence of the 5’UTR of the Cfp mRNA captured by 5’RACE highlighted in yellow. (TIF) [file pone.0184458.s002.tif]
